# From pairs of most similar sequences to phylogenetic best matches

**DOI:** 10.1186/s13015-020-00165-2

**Published:** 2020-04-09

**Authors:** Peter F. Stadler, Manuela Geiß, David Schaller, Alitzel López Sánchez, Marcos González Laffitte, Dulce I. Valdivia, Marc Hellmuth, Maribel Hernández Rosales

**Affiliations:** 1 Bioinformatics Group, Department of Computer Science, and Interdisciplinary Center for Bioinformatics, Universität Leipzig, Härtelstraße 16–18, 04107 Leipzig, Germany; 2 Competence Center for Scalable Data Services and Solutions Dresden/Leipzig, Interdisciplinary Center for Bioinformatics, German Centre for Integrative Biodiversity Research (iDiv), and Leipzig Research Center for Civilization Diseases, Universität Leipzig, Augustusplatz 12, 04107 Leipzig, Germany; 3 Max Planck Institute for Mathematics in the Sciences, Inselstraße 22, 04103 Leipzig, Germany; 4 Department of Theoretical Chemistry, University of Vienna, Währinger Straße 17, 1090 Vienna, Austria; 5 Facultad de Ciencias, Universidad National de Colombia, Sede Bogotá, Ciudad Universitaria, 111321 Bogotá, D.C. Colombia; 6 Santa Fe Institute, 1399 Hyde Park Rd., Santa Fe, NM87501 USA; 7 Software Competence Center Hagenberg GmbH, Softwarepark 21, 4232 Hagenberg, Austria; 8 School of Computing, University of Leeds, E C Stoner Building, Leeds, LS2 9JT UK; 9 CONACYT-Instituto de Matemáticas, UNAM Juriquilla, Blvd. Juriquilla 3001, 76230 Juriquilla, Querétaro, QRO México; 10 Departamento de Ingeniería Genética, Centro de Investigación y de Estudios Avanzados del IPN (CINVESTAV), Km. 9.6 Libramiento Norte Carretera Irapuato-León, 36821 Irapuato, GTO México

**Keywords:** Best matches, Gene tree, Species tree, Reconciliation, Orthology

## Abstract

**Background:**

Many of the commonly used methods for orthology detection start from mutually most similar pairs of genes (reciprocal best hits) as an approximation for evolutionary most closely related pairs of genes (reciprocal best matches). This approximation of best matches by best hits becomes exact for ultrametric dissimilarities, i.e., under the Molecular Clock Hypothesis. It fails, however, whenever there are large lineage specific rate variations among paralogous genes. In practice, this introduces a high level of noise into the input data for best-hit-based orthology detection methods.

**Results:**

If additive distances between genes are known, then evolutionary most closely related pairs can be identified by considering certain quartets of genes provided that in each quartet the outgroup relative to the remaining three genes is known. *A priori* knowledge of underlying species phylogeny greatly facilitates the identification of the required outgroup. Although the workflow remains a heuristic since the correct outgroup cannot be determined reliably in all cases, simulations with lineage specific biases and rate asymmetries show that nearly perfect results can be achieved. In a realistic setting, where distances data have to be estimated from sequence data and hence are noisy, it is still possible to obtain highly accurate sets of best matches.

**Conclusion:**

Improvements of tree-free orthology assessment methods can be expected from a combination of the accurate inference of best matches reported here and recent mathematical advances in the understanding of (reciprocal) best match graphs and orthology relations.

**Availability:**

Accompanying software is available at https://github.com/david-schaller/AsymmeTree.

## Background

The distinction of orthologous and paralogous pairs of genes, respectively, is of key importance in evolutionary biology as well as genome annotation. As defined by Walter Fitch [1, 2], two genes are orthologs if their last common ancestor (in the gene tree) corresponds to a speciation event, and they are paralogs if they arose through a duplication event. In general, orthologs are expected to have the same function in different organisms, while the functions of paralogs are usually similar but clearly distinct [3, 4].

A large class of computational approaches to orthology assessment [5, 6] uses symmetric best matches (SBM) [7], also known as bidirectional best hits (BBH) [8], reciprocal best hits (RBH) [9], or reciprocal smallest distance (RSD) [10]. The intuitive justification for these approaches is that symmetric best matches (in the sense of sequence similarity) approximate the idea of evolutionarily closest relatedness. These two concepts are not the same, however. The notion of evolutionary relatedness depends on the underlying phylogenetic tree *T* and is naturally expressed by comparing last common ancestors: a gene *x* is more closely related to a gene *y* than to $y'$ if the last common ancestor ${{\,\mathrm{lca}\,}}(x,y)$ is a successor of ${{\,\mathrm{lca}\,}}(x,y')$ in *T*.

**Fig. 1:**
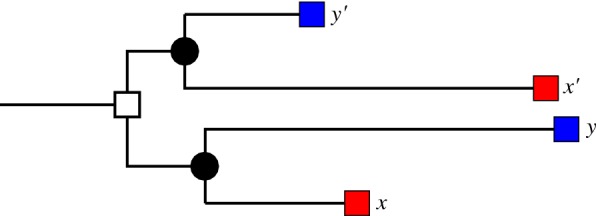
Lineage-specific rate variation between paralogs. The gene tree, with branch length indicating an additive evolutionary distances, pre-dates the speciation ($\bullet$) of the red and blue species. We have ${{\,\mathrm{lca}\,}}(x,y)\prec {{\,\mathrm{lca}\,}}(x,y')$ but $d(x,y')<d(x,y)$

From an evolutionary point of view, therefore, one is interested in *reciprocal best matches* (defined in terms of the gene tree *T*) rather than in *reciprocal best hits* (defined in terms of some distance of similarity measure between sequences). Best matches and best hits are equivalent if and only if the Molecular Clock Hypothesis is satisfied [11, 12]. In general this is not the case. In particular, paralogous members of a gene family often differ in their evolutionary rates due to (adaptive) changes in the function [13, 14]. Both the “Duplication-Degeneration-Complementation” (DDC) model [15] and the “Escape from Adaptive Conflict” (EAC) model [16] predict that the fate of paralogs, including their evolutionary rate, may differ substantially between lineages that diverge soon after the duplication event due to different selective pressures. The simplest case is shown in Fig. 1: an ancestral gene is duplicated before the speciation event leading to two species (indicated by colors), each containing two paralogs (denoted by *x* and $x'$ in the red species and *y* and $y'$ in the blue species). The two paralogs evolve with very different rates in the two species. Although *x* and y as well $x'$ and $y'$ are orthologs, the evolutionary rates are more similar between *x* and $y'$, and $x'$ and *y*, respectively. This situation is not at all uncommon. The asymmetric divergence of the genes in the HOXA cluster following the teleost-specific (3R) genome duplication may serve as a paradigmatic example [17]. While in fugu (*Takifugu rubripes*) and other percomorphs the HOXAb paralogs diverge faster, it is the HOXA13b paralog that evolves at a faster rate in zebrafish (*Danio rerio*), which diverged early from percomorphs within the Teleostei clade.

The situation as observed in the HOXA cluster is shown in Fig. 1. Here, the pair $x,y'$ shows the smallest evolutionary distance and hence will appear as reciprocal best hit, while the closest evolutionary relative of *x* is the gene *y*. This discrepancy is **not** a consequence of inaccurate measurements but an intrinsic feature of the evolutionary process: more evolutionary events have accumulated on the path from *x* to *y* than on the path from *x* to $y'$. The correct *reciprocal best hit* therefore does not coincide with the correct *reciprocal best match*. This immediately begs the question whether such cases can be detected from sequence comparisons. We consider this issue at two levels: (i) Can (reciprocal) best matches be identified *in principle*, i.e., from perfectly accurate data, and (ii) how well can this be done in practice? To address the first question we will assume that we can determine an additive distance between any two genes and investigate the consequences of this assumption. To investigate the accuracy that can be achieved from sequence data we will devise a simulation system to generate evolutionary scenarios with complex rate variations.

The focus on additive metrics is motivated by the close connection between additive metrics and evolutionary trees. More precisely, an additive metric determines a unique *unrooted* phylogenetic tree $\overline{T}$ as well as its branch length [18, 19], and *vice versa*. The determination of best matches, which are defined in terms of last common ancestors, however, requires a rooted phylogenetic tree *T*. From a theoretical point of view, therefore, the missing information is the placement of the root of *T* in the underlying unrooted phylogenetic tree $\overline{T}$.

The problem of determining the position of the root in an unrooted tree $\overline{T}$ has been well studied in the phylogenetic literature [20]. The most common approach is the inclusion of an outgroup, i.e., a taxon *z* known to branch earlier than the taxa of interest. The root is then located in the branch leading to *z*. Outgroup rooting can be unreliable in the presence of rapid radiations or when only very distant outgroups are available [21, 22]. The simplest method is midpoint rooting [23], which places the root at the midpoint on the longest path in the tree. Despite its simplicity it often works remarkably well [24]. An interesting variation on this theme is minimum variance rooting [25]. The estimation of dated phylogenies using a relaxed clock assumption yields an estimate for the position of the root as a by-product [26]. A related Bayesian method was introduced in [27]. In a phylogenomics setting, the root of the species tree can also be obtained by minimizing the number of inferred gene duplications [28]. Most recently, non-reversible substitution models have been employed for estimating rooted phylogenetic trees [29, 30].

From a practical point of view, furthermore, we wish to avoid the explicit construction of a (rooted or unrooted) gene tree *T* since reconstructing accurate evolutionary trees from individual gene sequences is a notoriously difficult problem. Instead we aim to stay as close as possible to the idea of reciprocal best hit methods and thus we will attempt to use only “local” comparisons of as few as possible measurements of evolutionary distances. This idea naturally leads us to considering quartets, i.e., unrooted trees describing four taxa, and the corresponding rooted triples. It is well known that the rooted triples are sufficient to determine the rooted tree in which they reside. Moreover, there is a polynomial-time algorithm that either constructs a rooted tree *T* from a set of rooted triples or determines that no such tree exists [31]. By Buneman’s Theorem [18, 19], an unrooted tree can be uniquely recovered from all its quartets. However, the problem of determining whether a given set is compatible (i.e., whether there is an unrooted tree $\overline{T}$ that contains all quartets) is NP-complete [32], a fact that reinforces the desire to avoid the explicit reconstruction of $\overline{T}$. Nevertheless, these classical results ensure that the relevant information is contained in quartets. More directly, we will show in this contribution that if we can reliably determine a suitable outgroup, best matches can be extracted from a small set of quartets.

Although much of this work is based on the assumption that an additive distance between taxa is available, one has to keep in mind that additive evolutionary distances, like divergence times, cannot be measured directly. While it is common practice to determine a dissimilarity $d'(x,y)$ of two taxa (genes) *x* and *y* from pairwise alignments, $d'$ is a systematic under-estimate of the number of events *d* due to back-mutations, and thus not additive. In practice, the conversion of measurements of $d'$ into an additive distance *d* that quantifies the number of evolutionary events is based on a Markov model of the evolutionary process. For sequence data, this may be the Jukes-Cantor model [33] or one of its more elaborate variants [34–36]. In the most benign setting, *d* and $d'$ are related by a monotone transformation, which in particular implies that the measured distances $d'$ correctly identify the best hits. It can also be shown, however, that non-additive distances in general cannot identify the correct topology of quartets [37]. Hence, we have no hope of computing correct best matches directly from non-additive (dis)similarities.

This contribution is organized as follows: in the following section we give a rigorous mathematical rendering of the background outlined above and use it to show that, given an additive distance measure, it is indeed possible to perfectly identify all best matches of a gene *x* of species *s* among its homologs $\{y_1,\dots ,y_k\}$ in species *t* provided a suitable outgroup *z* can be found for every set $\{x,y_i,y_j,z\}$ of four genes. As a consequence, the practical problem becomes the reliable identification of correct “relative outgroups”. Assuming knowledge on the phylogeny of species from which the genes are taken, we proceed to derive several conditions under which *z* cannot be a correct choice and use these insights to devise a heuristic approach that works nearly perfect given additive distance data. We then introduce (in “Methods” section) a simulation environment for generating gene family histories with complex rate variations and show that it is possible to recover best matches more accurately than approximating them by best hits.

## Theory

### Notation and basic definitions

Let *T* be a phylogenetic (gene) tree with leaf set *L*. For each gene $x\in L$ we denote by $\sigma (x)$ the species within which it resides. We write $L[s]=\{y\in L\mid \sigma (y)=s\}$ for the set of genes in species *s*. For a leaf set $L'\subseteq L$ we define the rooted tree $T[L']$ as the tree obtained from *T* by retaining only the vertices and edges along paths from the root to a leaf in $L'$ and suppressing all vertices with degree 2. The vertex set of a rooted tree *T* is endowed with a partial order $\prec$ such that $x\preceq y$ whenever *y* lies along the unique path connecting *x* and the root $\rho _T$. Thus the leaves are the minimal elements w.r.t. $\prec$. Furthermore, for $A \subseteq L$ we define the *last common ancestor*${{\,\mathrm{lca}\,}}(A)=\min \{z\mid x\preceq z \text { for all } x\in A\}$, where the minimum is taken w.r.t. the partial order $\prec$. Moreover, if $A=\{x,y\}$ contains only two elements, we write ${{\,\mathrm{lca}\,}}(x,y)$ instead of ${{\,\mathrm{lca}\,}}(\{x,y\})$. For every $u\in V(T)$, we denote by *T*(*u*) the subtree of *T* rooted at *u*.

Consider a gene *x* in species *s*. Among all genes in species $t\ne s$, the best matches of *x* are all those genes *y* in species *t* that have the lowest ${{\,\mathrm{lca}\,}}(x,y)$. These *y* are the closest relatives of *x* in species *t*. This concept is made precise in

#### **Definition 1**

[38]    Let *T* be a phylogenetic tree with leaf set *L* (denoting genes) and $\sigma :L\rightarrow {\mathscr {S}}$ identifying the species $\sigma (x)\in {\mathscr {S}}$ in which a gene *x* resides. Then $y\in L$ is a *best match* of $x\in L$, in symbols $x\mathrel {{\varvec{\rightarrow }}}y$, if ${{\,\mathrm{lca}\,}}(x,y)\preceq {{\,\mathrm{lca}\,}}(x,y')$ holds for all leaves $y'$ from species $\sigma (y')=\sigma (y)$.

The best match relation $\mathrel {{\varvec{\rightarrow }}}$ is reflexive (since ${{\,\mathrm{lca}\,}}(x,x)=x$), but it is neither transitive nor symmetric. Its mathematical properties are discussed in detail in [38, 39]. In particular, all orthologs of *x* are among its best matches [40].

The evolutionary relatedness of two taxa *x* and *y* is most directly expressed by the divergence time $\tau (x,y)$, which is the total time elapsed in both lineages since the last common ancestor of *x* and *y*. Here, we consider only the case that all leaves refer to extant genes or taxa, i.e., $\tau (x,y)=2{\hat{\tau }}({{\,\mathrm{lca}\,}}(x,y))$, where ${\hat{\tau }}$ is the age of ${{\,\mathrm{lca}\,}}(x,y)$. Divergence times are ultrametric by definition. Furthermore, there is a well-known one-to-one correspondence between isomorphism classes of dated, rooted, phylogenetic trees and ultrametrics, cf. [41, 42]. The best match relation $\mathrel {{\varvec{\rightarrow }}}$ can thus also be defined in terms of divergence time: $x\mathrel {{\varvec{\rightarrow }}}y$ if and only if

1 $$\eqalign{ y \in & \arg \min \tau \left( {x,y'} \right) \cr & y' \in L\left[ {\sigma \left( y \right)} \right] \cr}$$

The distinction between best hits and best matches thus is simply the distance function: best matches require divergence times, while best hits use one of several (dis)similarity measures for sequence data. They are equivalent under the Molecular Clock Hypothesis, which however fails for most real life data sets.

### Reconciliation of gene tree and species tree

Since genes evolve as part of species, we can expect that *a priori* knowlege of the species phylogeny can be helpful for understanding the phylogeny of a gene family. This link is made precise by considering the embedding of a gene tree *T* into a species tree *S*.

As it is possible that gene duplications and losses predate the first speciation event, we model the species tree *S* with leaf set ${\mathscr {S}}$ as a *planted* tree, i.e., we introduce a vertex $0_S$ that is called the *planted* root of *S* and has the “conventional” root $\rho _S={{\,\mathrm{lca}\,}}(L)$ as its single child. Using this construction, the embedding of the gene tree into the species tree is formalized by the *reconciliation map*$\mu : V(T)\rightarrow V(S)\cup E(S)$, which maps duplications to the edges of *S* and speciations to the inner vertices $V^0(S)$ of the species tree. We follow here the notation of [40]. Restricting ourselves to duplication/loss scenarios, i.e., disregarding horizontal gene transfer, the reconciliation map satisfies the root constraint (R0) $\mu (x) = 0_S$ if and only if $x = 0_T$; the leaf constraint (R1) $\mu (x)=\sigma (x)$ for $x\in L(T)$, the ancestor preservation (R2), i.e., $x\prec _{T} y \implies \mu (x)\preceq _S \mu (y)$, and the following two speciation constraints for all speciation vertices $\mu (x)\in V^0(S)$: (R3.i) $\mu (x) = {{\,\mathrm{lca}\,}}_S(\mu (v'),\mu (v''))$ for at least two distinct children $v',v''$ of *x* in *T*. (R3.ii) $\mu (v')$ and $\mu (v'')$ are incomparable in *S* for any two distinct children $v'$ and $v''$ of *x* in *T* [40]. Equivalent axiom systems are considered e.g. in [43–45]. Such reconciliation maps satisfy

2 $$\begin{aligned} \mu (x)\succeq _S {{\,\mathrm{lca}\,}}_S(\sigma (L(T(x)))), \end{aligned}$$

i.e, an event $x\in V(T)$ in the gene tree cannot be mapped to a node in the species tree below the last common ancestor of all the species In this contribution we assume that $\mu$ in addition satisfies (R4): If $\mu ({{\,\mathrm{lca}\,}}_T(x,y))=\mu ({{\,\mathrm{lca}\,}}_T(x,z))\in V^0(S)$, then ${{\,\mathrm{lca}\,}}_S(\sigma (x),\sigma (y))={{\,\mathrm{lca}\,}}_S(\sigma (x),\sigma (z))$. In essence, (R4) ensures that a single node in *T* cannot represent two distinct speciation events, i.e., that the gene tree *T* is not “less resolved” than the species tree *S* into which it is embedded.

The reconciliation map $\mu$ defines *event labels* on the inner nodes of the gene tree *T*, identifying *u* as a duplication node if $\mu (u)\in E(S)$ and as speciation if $\mu (u)\in V(S)$. While it is possibly to find a reconciliation map $\mu$ for every pair of gene and species tree [46], this is no longer true when event labels on *T* are given [45, 47]. Conversely, *T* and *S* imply constraints on the event labels, identifying nodes that have to be duplications under *any* reconciliation map [40]. Here, we characterize these nodes further. We start from the following technical results:

#### **Lemma 2**

([40, Lemma 2]) *LetT be a gene tree,Sbe a species tree and*$\mu :V(T)\rightarrow V(S)\cup E(S)$*be a reconciliation map without horizontal gene transfer that does not necessarily satisfy (R4)*. *Let*$x\in V(T)$*be a vertex with*$\mu (x)\in V^0(S)$. *]Then*, $\sigma (L(T(v')))\cap \sigma (L(T(v''))) = \emptyset$*for all distinct*$v',v''\in \mathsf {child}(x)$.

Let us first consider the case of binary gene trees:

#### **Lemma 3**

*LetT be a binary gene tree,S be a species tree, and*$\mu :V(T)\rightarrow V(S)\cup E(S)$*be a reconciliation map without horizontal gene transfer that does not necessarily satisfy (R4)*. *Let*$x,y\in L(T)$*be two genes with*$\sigma (x)\ne \sigma (y)$. *If*${{\,\mathrm{lca}\,}}_S(\sigma (x),\sigma (y))\prec \mu ({{\,\mathrm{lca}\,}}_T(x,y))$, *then*${{\,\mathrm{lca}\,}}_T(x,y)$*is a duplication event*.

#### *Proof*

Assume for contradiction $u:={{\,\mathrm{lca}\,}}_T(x,y)$ is a speciation event, i.e., $\mu (u)\in V^0(S)$. Let $v'$ and $v''$ be the two children of *u* in *T*. Observe that $u:={{\,\mathrm{lca}\,}}_T(x,y)$ implies that $x\in L(T(v'))$ and $y\in L(T(v''))$ or *vice versa*. W.l.o.g. we assume that $x\in L(T(v'))$ and $y\in L(T(v''))$. By (R3.i) and (R3.ii), $\mu (u) = {{\,\mathrm{lca}\,}}_S(\mu (v'),\mu (v''))$ and, in particular, $\mu (v')$ and $\mu (v'')$ are incomparable in *S*. Then by Lemma 2, we have $\sigma (L(T(v')))\cap \sigma (L(T(v''))) = \emptyset$. This and R2 implies that $\mu (v')\succeq _S\sigma (x)$ and $\mu (v')\succeq _S\sigma (y)$. The latter two arguments imply that ${{\,\mathrm{lca}\,}}_S(\sigma (x),\sigma (y))=\mu (u)$; a contradiction. $\square$

**Fig. 2:**
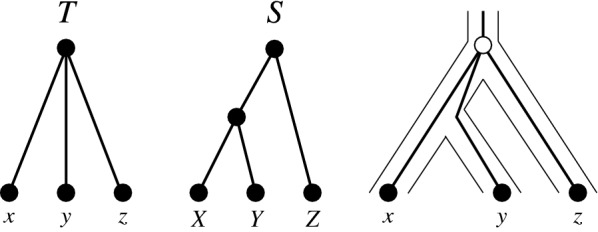
The reconciliation $\mu$ with $\mu ({{\,\mathrm{lca}\,}}_T(x,y,z))={{\,\mathrm{lca}\,}}_S(X,Y,Z)$ satisfied (R1), (R2), (R3.i), and (R3.ii) but does not admit an unambiguous interpretation of ${{\,\mathrm{lca}\,}}_T(x,y,z)$ as single event: it confounds the speciation separating *Z* and ${{\,\mathrm{lca}\,}}_S(Y,X)$ with a gene duplication leading the ancestor of *x* and *y* or with the speciation separating *X* and *Y*. In either interpretation, the reconciliation map $\mu$ does not correspond to a mechanistic explanation of the gene family history

The assumption that *T* is binary is necessary here as the example in Fig. 2 shows. Such reconciliations, however, cannot be meaningfully interpreted in terms of evolutionary events. Instead, the root of *T* confounds the duplication leading to *x* and *y* and the speciation separating ${{\,\mathrm{lca}\,}}_S(\sigma (x),\sigma (y))$ from $\sigma (z)$. To suppress such undesirable cases, we in addition require that $\mu$ satisfies axiom (R4). In essence, (R4) forbids to map two distinct speciation events to the same vertex of *S*.

#### **Lemma 4**

*Let*$\mu :V(T)\rightarrow V(S)\cup E(S)$*be a reconciliation map without horizontal gene transfer that satisfies (R4) and let*$x,y\in L(T)$*be two genes with*$\sigma (x)\ne \sigma (y)$. *If*${{\,\mathrm{lca}\,}}_S(\sigma (x),\sigma (y))\prec \mu ({{\,\mathrm{lca}\,}}_T(x,y))$, *then*${{\,\mathrm{lca}\,}}_T(x,y)$*is a duplication event*.

#### *Proof*

We assume that *T* is non-binary since the binary case is covered already by Lemma 3. Moreover, we assume, for contradiction, that $u:={{\,\mathrm{lca}\,}}_T(x,y)$ is a speciation event, i.e., $\mu (u)\in V^0(S)$. Let $v_x$ and $v_y$ be the children of *u* with $x\preceq _T v_x$ and $y\preceq _T v_y$; thus we have $\sigma (x)\in \sigma (L(T(v_x)))$ and $\sigma (y)\in \sigma (L(T(v_y)))$. Since $u={{\,\mathrm{lca}\,}}_T(x,y)$, $v_x$ and $v_y$ are incomparable in *T* and hence $v_x\ne v_y$. By (R3.i), $\mu (v_x)$ and $\mu (v_y)$ are incomparable in *S*. Lemma 2 implies $\sigma (L(T(v')))\cap \sigma (L(T(v''))) = \emptyset$ for all distinct children $v'$ and $v''$ of *u*. The latter two facts together with (R2) imply ${{\,\mathrm{lca}\,}}_S(\sigma (x),\sigma (y)) = {{\,\mathrm{lca}\,}}_S(\mu (v_x),\mu (v_y))\prec \mu (u)$. By (R3.i), $\mu (u) = {{\,\mathrm{lca}\,}}_S(\mu (v'),\mu (v''))$ for some children $v'$ and $v''$ of *u*, and thus ${{\,\mathrm{lca}\,}}_S(\mu (v'),\mu (v'')) = {{\,\mathrm{lca}\,}}_S(\sigma (z'),\sigma (z''))$ for some leaves $z'\in L(T(v'))$ and $z''\in L(T(v''))$ from different species $\sigma (z')\ne \sigma (z'')$.

We proceed by showing that for at least one of $\sigma (z')$ and $\sigma (z'')$ we have ${{\,\mathrm{lca}\,}}_S(\sigma (x),\sigma (z')) = {{\,\mathrm{lca}\,}}_S(\sigma (z'),\sigma (z''))$ or ${{\,\mathrm{lca}\,}}_S(\sigma (x),\sigma (z'')) = {{\,\mathrm{lca}\,}}_S(\sigma (z'),\sigma (z''))$. Suppose that ${{\,\mathrm{lca}\,}}_S(\sigma (x),\sigma (z')) \ne {{\,\mathrm{lca}\,}}_S(\sigma (z'),\sigma (z''))$. Hence, ${{\,\mathrm{lca}\,}}_S(\sigma (x),\sigma (z')) \prec _S {{\,\mathrm{lca}\,}}_S(\sigma (z'),\sigma (z'')) = \mu (u)$. Therefore, ${{\,\mathrm{lca}\,}}_S(\sigma (x),\sigma (z'')) = {{\,\mathrm{lca}\,}}_S(\sigma (z'),\sigma (z''))$. Similarily, if ${{\,\mathrm{lca}\,}}_S(\sigma (x),\sigma (z'')) \ne {{\,\mathrm{lca}\,}}_S(\sigma (z'),\sigma (z''))$, then ${{\,\mathrm{lca}\,}}_S(\sigma (x),\sigma (z')) = {{\,\mathrm{lca}\,}}_S(\sigma (z'),\sigma (z''))$. Hence, assume w.l.o.g. that ${{\,\mathrm{lca}\,}}_S(\sigma (x),\sigma (z')) = {{\,\mathrm{lca}\,}}_S(\sigma (z'),\sigma (z'')) \ne {{\,\mathrm{lca}\,}}_S(\sigma (x),\sigma (y))$ Now, by contraposition of (R4), we have $\mu (u) = \mu ({{\,\mathrm{lca}\,}}_T(x,y))\ne \mu ({{\,\mathrm{lca}\,}}_T(x,z')) = \mu (u)$; a contradiction. $\square$

Lemma 4 conveniently generalizes to sets of genes:

#### **Corollary 5**

*Let*$\mu :V(T)\rightarrow V(S)\cup E(S)$*be a reconciliation map without horizontal gene transfer that satisfies (R4) and let*$A\subseteq L(T)$*with*$|\sigma (A)|\ge 2$. *If*${{\,\mathrm{lca}\,}}_S(\sigma (A))\prec \mu ({{\,\mathrm{lca}\,}}_T(A))$, *then*${{\,\mathrm{lca}\,}}_T(A)$*is a duplication event*.

#### *Proof*

Note first that ${{\,\mathrm{lca}\,}}_T(A) = {{\,\mathrm{lca}\,}}_T(x,y)$ for some $x,y\in A$. Assume first $\sigma (x)\ne \sigma (y)$. Thus, ${{\,\mathrm{lca}\,}}_S(\sigma (A))\prec \mu ({{\,\mathrm{lca}\,}}_T(A))$ implies ${{\,\mathrm{lca}\,}}_S(\sigma (x),\sigma (y)) \preceq {{\,\mathrm{lca}\,}}_S(\sigma (A)) \prec \mu ({{\,\mathrm{lca}\,}}_T(A))=\mu ({{\,\mathrm{lca}\,}}_T(x,y))$. Hence, the statement follows from Lemma 4. If $\sigma (x)=\sigma (y)$, then ${{\,\mathrm{lca}\,}}_T(A) = {{\,\mathrm{lca}\,}}_T(x,y)$ implies that there are distinct children $v_x$ and $v_y$ of ${{\,\mathrm{lca}\,}}_T(A)$ with $v_x\succeq x$ and $v_y\succeq y$. Thus, ${{\,\mathrm{lca}\,}}_T(A) = {{\,\mathrm{lca}\,}}_T(v_x,v_y)$. However, since $\sigma (x)=\sigma (y)$ we have $\sigma (L(T(v_x)))\cap \sigma (L(T(v_y)))\ne \emptyset$. Thus, Lemma 2 implies that $\mu ({{\,\mathrm{lca}\,}}_T(A))\notin V^0(S)$ and hence, ${{\,\mathrm{lca}\,}}_T(A)$ is duplication. $\square$

### Trees and (dis)similarities

Neither the divergence times nor the ${{\,\mathrm{lca}\,}}$ function of the phylogenetic tree *T* can be measured directly. The next-best choice is to work with an evolutionary distance, which measures the number of evolutionary events that have taken place to separate two taxa. For each edge $e=uv$ in *T* it is given by $\ell (e)=\int _{{\hat{\tau }}(u)}^{{\hat{\tau }}(v)}\mu _e(t)dt$, where $\mu _e(t)$ is the rate of evolution. In general $\mu _e(t)$ depends both on the lineage, and thus the individual edges in *T*, as well as on the exact point in time along *e*. It associates with each edge *e* a measure $\ell (e)$ of changes incurred, and thus an additive distance. If $\mu _e(t)=\mu _0$ is constant, we simply have $d_{\ell ,T}(x,y)=\mu _0 \tau (x,y)$. This is the well-known Molecular Clock Hypothesis [11, 12].

In general, we consider $\ell : E(T)\rightarrow {\mathbb {R}}^+$ simply as an assignment of positive lengths to the edges of *T*, which we interpret as a measure proportional to the number of evolutionary events. This gives rise to a metric distance function $d_{\ell ,T}(x,y)$ on *L* defined as the sum of the lengths $\ell (e)$ of the edges *e* along the unique path connecting *x* and *y* in *T*. From *T* we obtain an associated *unrooted* tree $\overline{T}$ by (i) omitting the planted root $0_T$ and its incident edge, and (ii), in case the root $\rho$ in *T* has exactly two children $u_1$ and $u_2$, by replacing the path $u_1\rho u_2$ by a single edge $u_1u_2$ with length $\ell (u_1u_2):=\ell (u_1\rho )+\ell (\rho u_2)$. Note that the dissimilarity function $\ell$ is by construction the same on *T* and $\overline{T}$. Thus $\overline{T}$ determines *T* up to the position of the root, i.e., *T* is obtained from $\overline{T}$ by inserting the root into an edge of $\overline{T}$ or declaring an inner vertex of $\overline{T}$ as the root. As for rooted trees, we define the restriction $\overline{T}[L']$ for some subset $L'\subseteq L$ by retaining only the vertices and edges along the paths between pairs of vertices in $L'$ and then suppressing all vertices of degree 2. We note that $\overline{T[L']}=\overline{T}[L']$.

A dissimilarity *d* on *L* is called *additive* if there is an unrooted tree $\overline{T}$ with edge lengths $\ell$ such that $d=d_{\ell ,\overline{T}}$. A key result in mathematical phylogenetics [18, 19] characterizes additive (pseudo)metrics as those that satisfy the *four point condition*. It states that *d* is additive if and only if the restriction of *d* to each subset $L'$ of *L* with $|L'|=4$, usually called a *quartet*, is additive and thus determines a tree on four leaves. Furthermore, the unrooted tree $\overline{T}$ is uniquely defined by *d*. In principle, therefore, distance data completely determines a phylogenetic tree up to the position of the root.

The results of [18, 19] furthermore imply that $\overline{T}$ can be expressed in terms of its four-taxa subtrees. This provides us with a natural possibility to consider only “local” topologies instead of having to construct the unrooted tree $\overline{T}$ explicitly. To this end, we consider the restrictions $\overline{T}[p,q,r,s]$ of $\overline{T}$ to four distinct leaves $p,q,r,s\in L$ and define the *quartet relation* [48, 49] (*pq*|*rs*) if there is an edge *e* in $\overline{T}$, and thus in $\overline{T}[p,q,r,s]$, such that $\{p,q\}$ and $\{r,s\}$ are in different connected components of the forest obtained by removing *e* from $\overline{T}$ or $\overline{T}[p,q,r,s]$. Equivalently, we have [48, 49]

3 $$\begin{aligned} \begin{aligned} (pq|rs) \iff&d(p,q)+d(r,s) < \\&\min {( d(p,r)+d(q,s), d(p,s)+d(q,r) )}\,. \end{aligned} \end{aligned}$$

For additive metrics, the two distance sums on the second line are equal [18, 19]. All three terms are equal if and only if the four points form a star, whence the existence of a separating edge requires the strict inequality. By a slight abuse of notation we write $\overline{T}[p,q,r,s]=(pq|rs)$ if Eq. (3) holds, and $\overline{T}[p,q,r,s]={\varvec{\times }}$ if no quartet exists on these four leaves, i.e., if $\overline{T}[p,q,r,s]$ is the star tree.

### From quartets to rooted triples

In a *planted phylogenetic treeT* with leaf set $L\cup \{0_T\}$ all inner vertices have degree at least 3. The special leaf $0_T$ identifies the ancestral state. Its only neighbor is the root $\rho _T$. It will sometimes be useful to consider *T*(*u*) as planted tree by including the unique parent *v* of *u* and the edge *vu*. The leaf set of *T*(*u*) will be denoted by *L*(*T*(*u*)).

The most common method to specify the root of a phylogenetic tree is the use of so-called outgroups, that is, additional taxa that are known *a priori* to be outside a monophyletic group of interest. Given a planted (or rooted) phylogenetic tree, on the other hand, monophyletic groups are the leaf sets of a subtree, i.e., $L'$ is a monophyletic group if and only if there is a vertex $u\in V(T)$ such that $L'=L(T(u))$. Every leaf $x\in L\setminus L'$ is an outgroup for $L'$.

Every edge in an unrooted tree $\overline{T}$ defines a split $L'|L''$ of *L*, where $L'$ and $L''$ are the leaves in the connected components of  . At most one of the two subtrees $\overline{T'}$ and $\overline{T''}$ contains the root of the underlying phylogenetic tree *T*. If the root is not contained in $\overline{T'}$, then the tree $T'\cup \{e\}$ planted at the endpoint of *e* describes a monophyletic group. In this case all $x\in L''$ are outgroups for $T'$. Which subtrees of $\overline{T}$ correspond to monophyletic groups is determined by the position of the root, and therefore requires external information.

It will be convenient in the following to define outgroups not only for monophyletic groups.

#### **Definition 6**

For a phylogenetic tree *T* with leaf set *L*, consider a subset $L'\subseteq L$ and a leaf $z\in L\setminus L'$. We say that *z* is an *outgroup* for $L'$ if ${{\,\mathrm{lca}\,}}(L')\prec {{\,\mathrm{lca}\,}}(L',z)$.

**Fig. 3:**
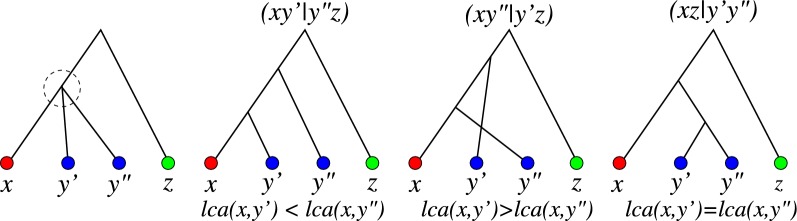
Relation of last common ancestors ${{\,\mathrm{lca}\,}}(x,y')$ and ${{\,\mathrm{lca}\,}}(x,y'')$, resp., with quartets on $\{x,y',y'',z\}$ with a trusted outgroup *z*

Let us now return to the quartets of $\overline{T}$. The following simple result, illustrated in Fig. 3, shows that quartets can be used to infer inequalities between ${{\,\mathrm{lca}\,}}$ vertices in *T* provided one of the four leaves is known to be an outgroup for the other three:

#### **Lemma 7**

*Supposezis an outgroup for*$\{x,y',y''\}$*inT*. *If*$\overline{T}[x,y',y'',z]$*is fully resolved, then*

(i) $${{\,\mathrm{lca}\,}}(x,y')={{\,\mathrm{lca}\,}}(x,y'')$$*iff*$$\overline{T}[x,y',y'',z]=(xz|y'y'')$$,
(ii) ${{\,\mathrm{lca}\,}}(x,y')\prec {{\,\mathrm{lca}\,}}(x,y'')$*iff*$\overline{T}[x,y',y'',z]=(xy'|y''z)$, *and*
(iii) $${{\,\mathrm{lca}\,}}(x,y')\succ {{\,\mathrm{lca}\,}}(x,y'')$$*iff*$$\overline{T}[x,y',y'',z]=(xy''|y'z)$$.

*Otherwise*, $$\overline{T}[x,y',y'',z]={\varvec{\times }}$$*and*$${{\,\mathrm{lca}\,}}(x,y')={{\,\mathrm{lca}\,}}(x,y'')$$.

#### *Proof*

Since *z* is an outgroup by assumption, there are only three possible fully resolved rooted tree with $L=\{x,y',y'',z\}$, see Fig. 3. Each of these trees corresponds to a unique quadruple (annotated at the top). The relationship between ${{\,\mathrm{lca}\,}}(x,y')$ and ${{\,\mathrm{lca}\,}}(x,y'')$ is determined by the tree topology. The statement follows by inspecting the three cases. If ${\bar{T}}[x,y',y'',z]$ is not fully resolved, no quartet is defined on $\{x,y',y'',z\}$, i.e., ${\bar{T}}$ is the star tree and thus ${{\,\mathrm{lca}\,}}(x,y')={{\,\mathrm{lca}\,}}(x,y'')={{\,\mathrm{lca}\,}}(y',y'')$. $\square$

#### **Observation 8**

If $u'={{\,\mathrm{lca}\,}}(x,y')$ and $v'={{\,\mathrm{lca}\,}}(x,y'')$ for $x,y',y''\in L$, then $u'$ and $v'$ are comparable w.r.t. $\preceq$ in *T*.

Lemma 7 together with Obs. 8 implies that quartets with known outgroups can be used to identify best matches. More precisely, in order to determine the set $\{y\in L[s] \mid x\mathrel {{\varvec{\rightarrow }}}y\}$ it suffices to consider leaf sets $\{x,y',y'',z\}$ with $y',y''\in L[s]$ such that *z* is an outgroup for $\{x,y',y''\}$. By Lemma 7, any set of this type implies an (in)equality between ${{\,\mathrm{lca}\,}}(x,y')$ and ${{\,\mathrm{lca}\,}}(x,y'')$. It may not be necessary to consider all quartets. To explore ways to reduce the computational efforts, let us assume that for given $x\in L$ and $s\in S$, $s\ne \sigma (x)$, we can identify sets $Y\subseteq L[s]$ and $Z\subseteq L$ such that the following three assumptions are satisfied:

(A0): The noise in the data is small enough so that for any four taxa $\{x,y',y'',z\}$ with $y',y''\in Y$ and $z\in Z$ one of the three possible quartets or the star topology is inferred correctly.
(A1): The candidate set $Y\subseteq L[s]$ contains all best matches of *x* in species *s* (but usually also additional leaves).
(A2): *Z* is a non-empty set of outgroups for $Y\cup \{x\}$.

Before we proceed, let us consider these three assumptions in some more detail. (A0) is satisfied by construction for additive distance data. In real-life applications it is often possible to obtain at least a very good approximation using explicit models of sequence evolution. In addition, several computational approaches have been proposed to estimate the quartet relation directly from sequence data. It is also worth noting that (A0) does not require precise distance data, it only asks for correct categorical data on the quartet relation.

Condition (A1) can always be enforced by setting $Y=L[s]$. We make this assumption explicit because in practice it will be desirable to work with small subsets $Y\subseteq L[s]$ as using *L*[*s*] may be too expensive for large gene families. The inclusion of very distant relatives may be problematic for the construction of good multiple sequence alignments and thus the extraction of the quartet relation. Furthermore, it may be difficult to find suitable outgroup data in this case. Thus we will limit *Y* to a manageable size and sufficient sequence similarity. In ProteinOrtho [50], for example, $Y\subseteq L[s]$ is defined as the set sequence with blast bit-scores exceeding a certain fraction of the best hit for *x* in species *s*.

Condition (A2), i.e., the knowledge of appropriate outgroups, is the only problematic assumption. As discussed above, distance-based methods by construction do not convey information on the root of the phylogenetic tree *T* but only determine its unrooted version $\overline{T}$. As a consequence, additional information, not contained in the pairwise distance measurements, is necessary to determine the edge in $\overline{T}$ that harbors the position of the root $\rho$ of *T* [51]. In general, *Z* will be chosen from one or more species that are outgroups to $\sigma (x)$ and *s* in *S*. Even if outgroup species are given, gene duplications may pre-date the divergence of the available species set, so that a given data set will usually violate (A2) for some pairs of leaves. We will return to these issues in more detail in the following sections. 
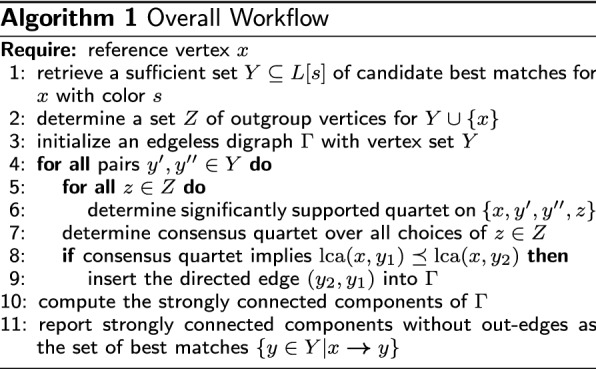


The discussion so far suggests to use the quadruple relation for sets of the form $\{x,y',y',z\}$ with $y',y''\in Y$ and $z\in Z$ to determine the best matches of *x* in the species containing the homolog set *Y*. The procedure is summarized in Alg. 1. The main result of this section establishes its correctness.

#### **Theorem 9**

*Algorithm 1 correctly identifies the set of best matches ofxwith colorsas the unique strongly connected component of* $\Gamma$*without out-edges provided assumptions (A0), (A1), and (A2) are satisfied.*

#### *Proof*

Assumptions (A1) and (A2) imply that comparison of the last common ancestors can be performed in terms of the quartets according to Lemma 7, which by assumption (A0) are all inferred correctly. Therefore, lines 4–7 compute all quartets correctly, and thus the inequality between ${{\,\mathrm{lca}\,}}(x,y_1)$ and ${{\,\mathrm{lca}\,}}(x,y_2)$ is inferred correctly. The auxiliary graphs $\Gamma$ therefore contains at least one arc between any two vertices $y',y''\in Y$ and both the arc $(y',y'')$ and $(y'',y')$ if and only if ${{\,\mathrm{lca}\,}}(x,y')={{\,\mathrm{lca}\,}}(x,y'')$, i.e., the strongly connected components are cliques. Since the ${{\,\mathrm{lca}\,}}(x,y)$ are interior vertices of *T* that are totally ordered along the path from *x* to the root of *T* (Observation 8), there is a unique strongly connected component *B* in $\Gamma$ that has no out-edges, whose vertices are those $y\in B$ for which ${{\,\mathrm{lca}\,}}(x,y)$ is minimal. Thus *B* is the set of best matches of *x* with color *s*. $\square$

Algorithm 1 therefore works correctly at least under idealized assumptions. It also serves as a heuristic in cases where one of the assumptions (usually (A2)) is violated.

### Identification of outgroups

In many practical applications, the phylogenetic relationships between the *species* under consideration are known. We therefore investigate here to what extent knowledge of the species tree *S* can help to identify good outgroup sets *Z*. Ideally, the genes chosen as outgroups *Z* are co-orthologs of the focal gene set *Y*, i.e., the duplication event that produced $y'$ and $y''$ occured after the speciation event that separates $\sigma (z)$ for all $z\in Z$ from $\sigma (X)$ and $\sigma (Y)$. As we shall see, it is not possible to identify outgroups with complete certainty. It is possible, however, to identify incorrect choices in many situations.

In the following we consider three species $\sigma (X)$, $\sigma (Y)$, and $\sigma (z)$ for $z\in Z$ such that

4 $$\begin{aligned} {{\,\mathrm{lca}\,}}_S(\sigma (X),\sigma (Y)) \prec _S {{\,\mathrm{lca}\,}}_S(\sigma (X),\sigma (Y),\sigma (z)), \end{aligned}$$

i.e., $\sigma (z)$ is an outgroup in the species tree for $\sigma (X)$ and $\sigma (Y)$. Problematic cases in which quartets are interpreted incorrectly may appear whenever the duplication event ${{\,\mathrm{lca}\,}}_T(y',y'')$ separating two paralogs $y',y''\in Y$ pre-dates the speciation event separating $\sigma (z)$ from ${{\,\mathrm{lca}\,}}_S(\sigma (X),\sigma (Y))$. We capture this situation in

#### **Definition 10**

Let *u* be an inner node of the species tree *S*, let $y',y''\in Y$ be paralogs in a species $\sigma (Y)\in L(S(u))$. Then ${{\,\mathrm{lca}\,}}_T(y',y'')$ is an *ancient duplication relative to*$u\in V(S)$ for the reconciliation map $\mu : V(T)\rightarrow V(S)\cup E(T)$ if $u \prec _S \mu ({{\,\mathrm{lca}\,}}_T(y',y''))$.

Clearly, if ${{\,\mathrm{lca}\,}}_T(y',y'')$ is an ancient duplication relative to ${{\,\mathrm{lca}\,}}_S(\sigma (X),\sigma (Y),\sigma (z))$, then genes in $z\in Z$ are bad choices as outgroups $\{x,y',y'',z\}$. The difficulty is that we do not know the reconciliation map $\mu$ in our setting. In some cases, however, it is possible to identify vertices in *T* that are ancient duplications relative to some speciation for *any* reconciliation. Such cases can then be avoided.

Before we investigate possibilities to identify some ancient duplications in distance data, we prove a rather technical result that shows that in cases without too many ancient duplications, Algorithm 1 produces correct results. For the proof we will need to consider the reconciliation map $\mu$ for *complete* gene family histories, i.e., gene trees *T* containing extant genes as well as all branches leading to loss events. As above, we do not consider HGT. The leaf set of *T* is thus  , where $L_e$ represents the extant genes and $L_0$ denotes loss events. Since the species map is naturally restricted to extant genes (i.e., $\sigma : L_e(T)\rightarrow L(S)$), we need to restrict (R1): If $x\in L_e(T)$, then $\mu (x)=\sigma (x)$. We will refer to such gene trees and reconciliation maps as *extended* gene trees and *extended* reconciliation maps, respectively. Correspondingly, Lemma 2 only holds for $L_e$, i.e., we can conclude that $\sigma (L_e(T(w_1)))\cap \sigma (L_e(T(w_2)))=\emptyset$. This can easily be seen by reusing the contradiction argument in [40][Lemma 2]. As a consequence of loss events we now may have $\sigma (L_e(T(v)))=\emptyset$ for some nodes $v\in V(T)$.

#### **Lemma 11**

*Let*$(T,\sigma )$*be an extended gene tree with a non-empty set of extant genes**with*$|\sigma (Z)|=1$, *letSbe a species tree on*$S=\{\sigma (X)$, $\sigma (Y)$, $\sigma (Z)\}$*such that*${{\,\mathrm{lca}\,}}_S(\sigma (X),\sigma (Y))\prec {{\,\mathrm{lca}\,}}_S(\sigma (X),\sigma (Y),\sigma (Z))=\rho _S$,*and let*$\mu$*be an extended reconciliation map from*$(T,\sigma )$*toS*. *If (A0) holds and*$|\mu ^{-1}(\rho _S)|\le 2$, *then Algorithm 1, usingYas the candidate best match set andZas outgroup set, correctly determines, for every gene*$x\in X$, *all best matches in species*$\sigma (Y)$.

#### *Proof*

First note that the statement is trivial if there exists only one gene in *Y*. Hence, we can assume that *Y* contains more than one gene. Moreover, Condition (A1) is trivially satisfied since, by assumption, the candidate set of best matches of *x* in *Y* is exactly *Y*. Since $L_e$ is non-empty, we have to consider the two cases $|\mu ^{-1}(\rho _S)|=1$ and $|\mu ^{-1}(\rho _S)|=2$.

Assume first $|\mu ^{-1}(\rho _S)|=1$, i.e., there exists exactly one $v\in V(T)$ such that $\mu (v)=\rho _S$. We then have $\sigma (L_e(T(w_1)))\cap \sigma (L_e(T(w_2)))=\emptyset$ for any distinct $w_1,w_2\in \mathsf {child}_T(v)$ (cf. [40] [Lemma 2], Lemma 2), which, by construction of the species tree *S*, immediately implies $\sigma (L_e(T(w)))\in \{\{\sigma (X),\sigma (Y)\},\{\sigma (Z)\}\}$ for any $w\in \mathsf {child}_T(v)$. Hence, *Z* is an outgroup set for $Y\cup \{x\}$, i.e., Condition (A2) is satisfied, and the statement thus follows directly from Theorem 9.

Now suppose $|\mu ^{-1}(\rho _S)|=2$, i.e., there are exactly two distinct $v_1,v_2\in V(T)$ with $\mu (v_1)=\mu (v_2)=\rho _S$. Let $T_1:=T(v_1)$ and $T_2:=T(v_2)$ be the subtrees of *T* rooted at $v_1$ and $v_2$, resp., and assume w.l.o.g. $x\in L_e(T_1)$. Note that $L_e(T_1)\cup L_e(T_2)=L_e$. Let $w_1\in \mathsf {child}_T(v_1)$ such that $x\preceq _T w_1 \prec _T v_1$. If $w_1$ were mapped to an edge or vertex along the path from $\rho _S$ to $\sigma (Z)$, then ${{\,\mathrm{lca}\,}}_S(\sigma (X),\sigma (Y))\prec {{\,\mathrm{lca}\,}}_S(\sigma (X),\sigma (Y),\sigma (Z))=\rho _S$ would imply $\sigma (X)\not \preceq _S \mu (w_1)$; a contradiction to (R2). Thus, $\sigma (Z)\notin \sigma (L_e(T(w_1)))$. Since $\mu (v_1)\in V^0(S)$, Condition (R3.i) implies that there exists $w_2\in \mathsf {child}_T(v_1)$, $w_2\ne w_1$, such that $\mu (v_1)={{\,\mathrm{lca}\,}}(\mu (w_1),\mu (w_2))$. Since $\sigma (L_e(T(w_1)))\cap \sigma (L_e(T(w_2)))=\emptyset$ by Lemma 2, we obtain $\sigma (L_e(T(w_1)))\subseteq \{\sigma (X),\sigma (Y)\}$ and $\sigma (L_e(T(w_2)))\subseteq \{\sigma (Z)\}$. We distinguish the two cases (a) $\sigma (Y)\notin \sigma (L_e(T_1))$ and (b) $\sigma (Y)\in \sigma (L_e(T_1))$.

*Case (a):* If $\sigma (Y)\notin \sigma (L_e(T_1))$, any leaf $y\in Y$ must reside within a subtree $T(w')$ with $w'\in \mathsf {child}_T(v_2)$, thus all genes in *Y* are best matches of *x*. Since the speciation node $v_2$ separates $\sigma (Z)$ from $\sigma (X)$ and $\sigma (Y)$, we have $\sigma (Z)\notin \sigma (L_e(T(w')))$ for any such $w'$ (cf. Lemma 2). Moreover, reusing the same arguments as for $v_1$, we conclude that there exists exactly one such $w'\in \mathsf {child}_T(v_2)$ such that $\sigma (Y)\in \sigma (L_e(T(w')))$. Hence, any two distinct $y,y'\in Y$ reside within the same subtree $T(w')$ and thus ${{\,\mathrm{lca}\,}}_T(x,y)={{\,\mathrm{lca}\,}}_T(x,y')$. Since $\sigma (Z)\notin \sigma (L_e(T(w')))$, this immediately implies $\overline{T}[x,y,y',z]=(xz|yy')$ for any $z\in Z$. Hence, $\Gamma$ is the complete graph, i.e., any gene of species $\sigma (Y)$ is correctly inferred as a best match of *x*.

*Case (b):* Assume, for contradiction, that there exists $w_3\in \mathsf {child}_T(v_1)\setminus \{w_1\}$ such that $\sigma (Y)\in \sigma (L_e(T(w_3)))$. Clearly, $w_3\ne w_2$. Since it must hold $\sigma (L_e(T(w_1)))\cap \sigma (L_e(T(w_3)))=\emptyset$ as well as $\sigma (L_e(T(w_2)))\cap \sigma (L_e(T(w_3)))=\emptyset$ by Lemma 2, we conclude $\sigma (L_e(T(w_1)))=\{\sigma (X)\}$ and $\sigma (L_e(T(w_3)))=\{\sigma (Y)\}$. However, (R4) then implies ${{\,\mathrm{lca}\,}}_S(\sigma (X),\sigma (Y))={{\,\mathrm{lca}\,}}_S(\sigma (Y),\sigma (Z))$; a contradiction. Hence, there exists an extant gene $y\preceq _T w_1$ in *Y*. Then, as $\sigma (Z)\notin \sigma (L_e(T(w_1)))$, any $z\in Z$ infers the same quartet on $\{x,y,y',z\}$, $y'\in Y\setminus \{y\}$. We therefore conclude that the auxiliary graph $\Gamma$ contains a unique strongly connected component without out-edges, which represents the set of best matches of *x* in *Y*. Note that in these cases Condition (A2) is not necessarily satisfied, but Algorithm 1 still provides the exact solution. $\square$

**Fig. 4:**
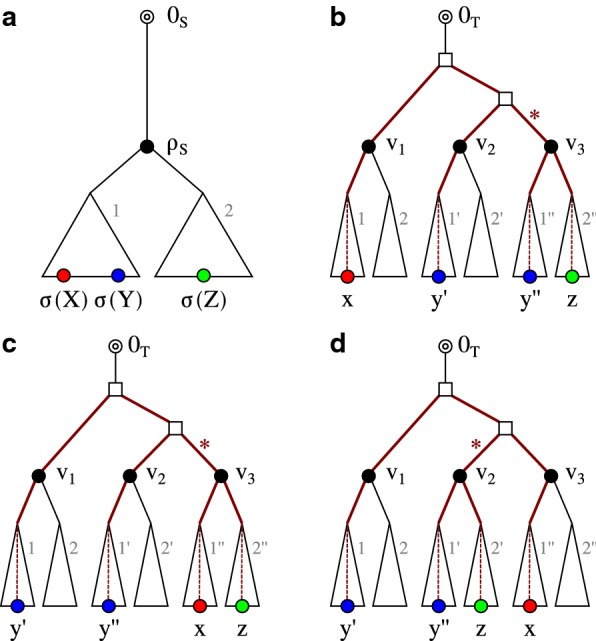
Minimal examples in which ancient duplications lead to false positives (FP) or false negatives (FN) when choosing outgroups as described in the text. (A) The species tree *S* displaying the triple ($\sigma (X)\sigma (Y)|\sigma (Z)$). (B-D) Gene trees *T* with two ancient duplications. We assume that $y'$ and $y''$ are the only extant genes of color $\sigma (Y)$, i.e. color $\sigma (Y)$ is extinct in the subtree of *x* in each of the shown cases. The asterisk marks the discriminating edge for the quartet inference. (B) A quartet $(xy'|y''z)$ is inferred so that only $y'$ but not $y''$ is a best match, $(x, y'')$ is a FN. (C) A quartet $(xz|y'y'')$ is inferred so that $y'$ is a false best match, $(x, y')$ is a FP. (D) A quartet $(xy'|y''z)$ is inferred so that $(x, y')$ is a FP and $(x, y'')$ is a FN

The condition $|\mu ^{-1}(\rho _S)|\le 2$ makes an explicit assumption on the true history of the gene family by limiting the scenario to at most one ancient duplication on  . Figure 4 shows that this condition cannot be dropped: if there are two or more ancient duplications affecting *X*, *Y*, and *Z*, then the correct inference of best matches from quartets can no longer be guaranteed. It is important to note that the condition $|\mu ^{-1}(\rho _S)|\le 2$ cannot be checked in real data since $\mu$ is unknown. In the simulated data, however, it is easy to validate and we observed empirically that it is rarely violated in our data (see “Simulation results” section).

**Fig. 5:**
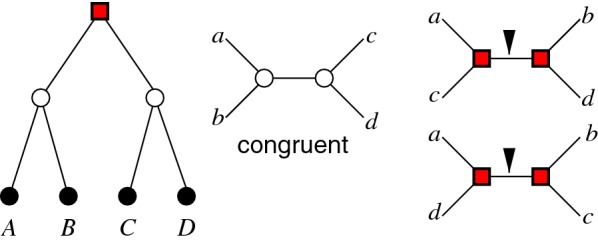
Incongruence of gene and species quartets implies the existence of an ancient duplication. Consider four pairwise distinct species *A*, *B*, *C*, and *D* whose species tree is given on the l.h.s., and let four genes *a*, *b*, *c*, and *d* be chosen such that $\sigma (a)=A$, $\sigma (b)=B$, $\sigma (c)=C$, and $\sigma (d)=D$. The two speciation events separating *A* from *B* and *C* from *D* are indicated by $\bigcirc$. The root of this tree is indicated by $\blacksquare$. Of the three possible gene quartets, one is congruent with the species tree. The other two are incongruent. In each of these, Eq. (2) implies that the two interior vertices in these quartets cannot be mapped to the species tree below the root. The root of the gene tree must thus be mapped above the root of the species tree

In some situations ancient duplications can be inferred unambiguously, independent of the reconciliation map $\mu$. This is in particular the case if there are incongruences between quartets of genes and species. Consider four genes *a*, *b*, *c*, *d* residing in four pairwise distinct species $\sigma (a)$, $\sigma (b)$, $\sigma (c)$, and $\sigma (d)$, and assume that these four species form the quartet $(\sigma (a)\sigma (b)|\sigma (c)\sigma (d))$. Then we say that the gene and species quartets are *congruent* if $\overline{T}[a,b,c,d]=(ab|cd)$ or $\times$. Otherwise, i.e., for $\overline{T}[a,b,c,d]\in \{ (ac|bd), (ad|bc)\}$, we say they are *incongruent*, see Fig. 5. In the following we show that the incogruence of gene and species quartets implies ancient duplications. More precisely:

#### **Theorem 12**

*Let*$$(T,\sigma )$$*andS be gene and species trees, respectively, and*$$a,b,c,d\in L(T)$$. *Moreover, let*$$\sigma (a)$$, $$\sigma (b)$$, $$\sigma (c)$$, *and*$$\sigma (d)$$*be pairwise distinct species, set*$$u:= {{\,\mathrm{lca}\,}}_S(\sigma (a),\sigma (b),$$$$\sigma (c),\sigma (d))$$, $$v_1:= {{\,\mathrm{lca}\,}}_S(\sigma (a),\sigma (b))$$, *and*$$v_2:= {{\,\mathrm{lca}\,}}_S(\sigma (c),\sigma (d))$$. *If*$$v_1\prec _S u$$, $$v_2\prec _S u$$*and*$$\overline{T}[a,b,c,d]=(ac|bd)$$*or*$$\overline{T}[a,b,c,d]=(ad|bc)$$, *then*$$u\prec _S \mu ({{\,\mathrm{lca}\,}}_T(a,b,c,d))$$*for every reconciliation map*$$\mu :V(T)\rightarrow V(S)\cup E(S)$$*without HGT events. In particular,*$${{\,\mathrm{lca}\,}}_T(a,b,c,d)$$*is a duplication event*.

#### *Proof*

By assumption, $S[\sigma (a),\sigma (b),\sigma (c),\sigma (d)]$ has the topology shown in Fig. 5. Assuming (*ac*|*bd*), Eq. (2) implies $\mu ({{\,\mathrm{lca}\,}}_T(a,c))\succeq {{\,\mathrm{lca}\,}}_S(\sigma (a),\sigma (c))=u$ and $\mu ({{\,\mathrm{lca}\,}}_T(b,d))\succeq {{\,\mathrm{lca}\,}}_S(\sigma (a),\sigma (c))=u$. Thus both inner nodes *p* and *q* of the quartet are mapped no lower than *u*. The edge between them therefore must be mapped to an edge pre-dating *u*, since the speciation constraint (R3) implies that two $\prec _T$-comparable events in *T* of which one is a speciation cannot by mapped to the same vertex of *S*. Thus $u \prec _S \mu ({{\,\mathrm{lca}\,}}_T(a,b,c,d))$. The case (*ad*|*bc*) is handled by an analogous argument exchanging *c* and *d*. The fact that ${{\,\mathrm{lca}\,}}_T(a,b,c,d)$ is a duplication event now follows from Lemma 4. $\square$

This theorem can be used to discard suspicious outgroups: If $\overline{T}[x,y,z_1,z_2]$ is incongruent with the known species tree, then $\sigma (z_1)\ne \sigma (z_2)$ should be replaced by outgroup candidates from earlier-branching species. The downside of using Theorem 12 is that it requires the systematic investigation of a possibly large numbers of quartets.

We suspect that it is possible in most cases to unambiguously identify pairs whose last common ancestor in the gene tree pre-dates the last common ancestor of the species tree under consideration. While it may be difficult to determine the relative order of such duplications, we suspect that clustering methods used to extract groups of co-orthologs (COGs) can be adapted to disentangle such ancient “paralog groups”.

## Simulation results

Well curated data for gene family histories are not available at large scale. We therefore use simulated data to evaluate how well best matches (in the sense of evolutionary relatedness) can be estimated from both perfect and noisy evolutionary distance measurements. For this purpose, it is important to have data sets that emphasize asymmetric rate variations among paralogs, i.e., the situations in which sequence dissimilarities and divergence times are not well correlated. We therefore developed a simulation system (see “Methods” section) that can produce this type of test data. Each *scenario* consists of a dated, planted species tree *S* and a gene tree *T*, that was simulated along *S* and thus is also dated. Each edge in *T* is assigned a rate, drawn from a distribution to model rate differences between paralog groups following gene duplications [14, 15, 52]. The product of the time difference between the end points of an edge and the evolutionary rate assigned to it then defines its length. The genetic distance *d*(*x*, *y*) of two genes *x* and *y* is the sum of the edge lengths along the unique path connecting *x* and *y* in *T*, see Eq. (7). Thus *d* is additive by construction. A typical example of a gene tree with distances can be found in Additional file 1: Fig. S1. In total, we simulated 2000 scenarios.

Since perfect additivity of *d* cannot be expected in the presence of measurement noise, we therefore superimposed normally distributed noise on the distance data, using the standard deviation *s* to control the noise level, see "Simulation of measurement noise” section. As a more realistic way to produce noisy data, we instead simulated sequence data along *T*, such that the expected number of events is proportional to the edge length, and thus to *d*.

Gene families in real-life data differ quite drastically from each other not only in their rate of sequence evolution but also as far as the rates of gene duplication and loss of paralogs in concerned. We therefore consider here a mix of scenarios with a different number of species. See Additional file 1: Figs. S2–5 for various statistics of the data set including the distribution of species and gene number per scenario, the average number of genes per species, and the distribution of edge lengths.

### Best matches from evolutionary distances

We compare three strategies to estimate best matches directly from the evolutionary distance *d*:

**1. Reciprocal best hits** are inferred directly from the distance data. In order to account for rate variations among paralogs, we follow the strategy of ProteinOrtho [50] and consider nearly co-optimal best hits by considering for a given gene *x* in species $\sigma (x)$ all those $y\in Y$ as almost best hits that have distance not worse than a factor $1+\epsilon$ than the most similar gene in *Y*. In symbols:

5 $$\begin{aligned} H(Y|x):= \{ y\in Y\mid d(x,y)\le (1+\epsilon )\min _{y'\in Y} d(x,y') \} \end{aligned}$$

**Fig. 6:**
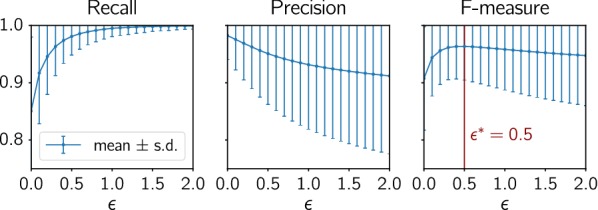
Recall, precision, and F-measure of the true best matches as a function of $\epsilon$ for simulated data (2000 scenarios)

For further comparison we then chose the value of $\epsilon ^*$ that maximizes the F-measure ($\epsilon ^*=0.5$, see Fig. 6). Still, this approach produces a substantial number of both false positives and false negatives in data sets with large rate variations between paralogs. We expect that the optimal value of $\epsilon ^*$ will depend on the details of the data set, in particular on the extent of evolution rate asymmetries. In general these will have to be estimated from the gene family history. We refer to this approach as the “$\epsilon$-method”. Since we chose the cut-off $\epsilon ^*$ to maximize the *F*-measure, we effectively determine an upper bound on the performance of the best hits approach.

**2. Explicit reconstruction of**$\overline{T}$. Since the additive distances completely determine $\overline{T}$, the only source of errors for perfect data is a potentially incorrect choice for the root of $\overline{T}$. For additive distance data, the Neighbor Joining algorithm [53] is guaranteed to produce the correct $\overline{T}$ [54]. We then use midpoint rooting [24] to pass from $\overline{T}$ to $T^{*}$ and compute the best matches in *T*. We refer to this method as “NJ+midpoint rooting”. This method is not intended as a viable means of analysis for real-life sequence data. It serves, however, as a convenient way to assess the effects of rate imbalances because it isolates the errors that are introduceεd by the choice of the root alone i.e., by rate imbalances.

**3.** The “***Quartet***” **approach** starts from a known (rooted) species tree *S*. For $x\in X$, and $y',y''\in Y$ we select the set of outgroup genes *Z* from outgroup species w.r.t. the species of *X* and *Y*, i.e., ${{\,\mathrm{lca}\,}}_S(\sigma (X),\sigma (Y))\prec {{\,\mathrm{lca}\,}}_S(\sigma (X),\sigma (Y),\sigma (z))$ for $z\in Z$. To reduce the risk for too many ancient duplications, which are a source of error in this approach (see Lemma 11), we may require in addition that ${{\,\mathrm{lca}\,}}_S(\sigma (X),\sigma (Y),\sigma (z))=\rho _S$, i.e., we only allow outgroup species from “the other side of the root”. For reasons of time complexity, we randomly select $\min (20,|{\tilde{Z}}|)$ among the genes ${\tilde{Z}}$ that meet this condition as the final outgroup set *Z* in Algorithm 1. Since we operate on distance data, quartets can directly be estimated using Eq. (3).

In order to benchmark the inference of best matches we compute recall and precision w.r.t. the true best matches restricted to pairs of gene sets *X* and *Y* for which such outgroups are available. On average we could assign outgroup genes to 74.6% of the $n(n-1)/2$ gene pairs, where *n* is the number of non-loss leaves in the respective scenario (see also Additional file 1: Fig. S6 for the scenario-wise percentage).

**Fig. 7:**
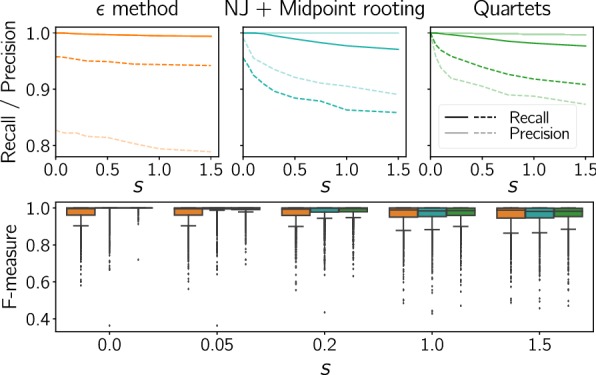
Performance comparison of the best match inference methods for simulated data (2000 scenarios). Top panel: Median (solid) and 10^th^ percentile (dashed) of recall and precision as a function of noise level *s* (standard deviation of the distribution from which perturbations were drawn, see “Simulation of measurement noise” section). Lower panel: Boxplots of F-measure for different levels of noise superimposed on the additive distance; $s=0$ refers to perfect data. Orange: $\epsilon$ method, turquoise: explicit construction of the unrooted tree $\overline{T}$ and midpoint rooting, green: inference of quartets with outgroups chosen in another branch of the root

The comparison in Fig. 7 (bottom panel) shows that the quartet method outperforms the alternatives for different levels of the simulated random measurement error in terms of F-measure. As an example, for $s=1.0$ the 10th percentile of the F-measure reaches 0.909 for the quartet method compared to 0.869 and 0.884 for the $\epsilon$-method and the Neighborjoining trees, respectively. The median values, on the other hand, are almost identical and fairly high (around 0.985). Hence, we suspect that more sophisticated methods are advantageous for a number of rare (but not negligible) difficult cases. Moreover, note that both recall and precision are almost perfect for noiseless data ($s=0$) and that the results are robust over a wide range of simulated measurement error. The same was observed for systematically biased noise (see Additional file 1: Fig. S7), which was simulated by computing a convex combination of the original matrix and a perturbation matrix derived from another tree. The performance of all three methods drops quickly when the perturbation becomes large.

The highest and most stable recall values could be obtained with the $\epsilon$-method for both types of noisy data. For our choice of $\epsilon$, this clearly comes at the cost of precision. Not surprisingly, the reconstruction of Neighborjoining trees already provides a higher precision than the $\epsilon$-method. The simple midpoint rooting strategy however still incurs noticable level of error. For the quartet method operating on noiseless data the only source of errors are bad choices of outgroups, which are the consequence of ancient duplications. The number of ancient duplication exceeds 1 in $5.15\%$ of the simulated gene family scenarios. Due to loss events predating the root of the species tree, the condition in Lemma 11 is only violated in $3.7\%$ of the gene trees. Out of these problematic cases, little more than half ($2.25\%$) actually result in a non-perfect inference accuracy.

**Fig. 8:**
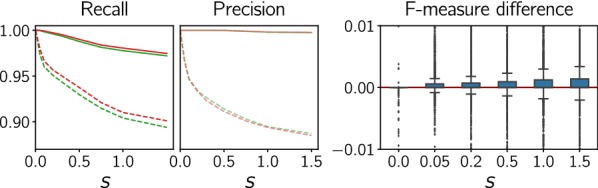
Performance comparison of two different outgroup choice methods: (1) outgroups chosen randomly from species in another branch of the root (green, same as in Fig. 7), (2) closest outgroups in all relative outgroup species corrected with Theorem 12 (red). Left and middle: Median (solid) and 10th percentile (dashed) of recall and precision as a function of noise level. Right: Boxplots of the F-measure differences for selected noise levels (method 2 − method 1)

Restricting the choice of outgroup genes *z* to species that are separated from *X* and *Y* by the root of the species tree, i.e., such that ${{\,\mathrm{lca}\,}}_S(\sigma (z),{{\,\mathrm{lca}\,}}_S(\sigma (X),\sigma (Y)))=\rho _S$, is likely to be problematic whenever *S* is skewed in a way that leaves very few choices for $\sigma (z)$ and whenever the divergence between $\sigma (z)$ and ${{\,\mathrm{lca}\,}}_S(\sigma (X),\sigma (Y))$ is large. In the latter case, saturation effects may impair the quartet inference in fast evolving gene families. Hence, it would be advantageous to consider also genes from closer species. In principle, every relative outgroup w.r.t. species $\sigma (X)$ and $\sigma (Y)$ is a viable candidate. These can then be filtered by applying Theorem 12 to reduce the number of bad choices of *z*. We find that filtering for outgroups with identifiable ancient duplications and giving preferences to the closest outgroup genes, i.e., those with the lowest ${{\,\mathrm{lca}\,}}_S(\sigma (z),{{\,\mathrm{lca}\,}}_S(\sigma (X),\sigma (Y)))$ indeed yields a further moderate improvement of the estimated best matches (see Fig. 8). However, the performance is slightly reduced for perfectly additive data due to ancient duplications that were not detected by the currently available filtering heuristics.

At first glance the F-measures in Figs. 7, 8 look “to good to be true”. Our collection of scenarios, however, contains many easy instances with few paralogs and losses. Real-life data furthermore are plagued by systematic biases, incomplete and missing annotations, inconsistent choices between isoforms, etc., that affect the ability to correctly estimate evolutionary distances and thus pairwise best hits. So far, we have assumed that we have perfect data in this respect and evaluate only our ability to recover (reciprocal) best matches. In the following we briefly consider the effect of having to estimate evolutionary distances from sequences. Again, we will consider only the most benign situation, i.e., sequences generated from Markov processes.

### Best matches from sequences

In applications to real-life data sets, additional uncertainties arise through the reconstruction of distances from sequences. We therefore simulated sequences (without in/dels) from the gene tree/species tree scenarios and inferred the best matches from the sequence data. Considering only substitutions avoids the need for computing sequence alignments. We explored two different ways of determining the quartet relation: (a) We derived an approximately additive evolutionary distance from the observed dissimilarity, before again applying Eq. (3). More precisely, for nucleic acid sequences we transformed the normalized Hamming distance using the simple Jukes-Cantor transform [33], and for amino acid sequences we applied the BLOSUM-based transformation [55] in the Biopython package [56]. (b) We directly inferred the quartets using the Quartet Mapping method (QM) [57] as outlined in “Methods” section.

**Fig. 9:**
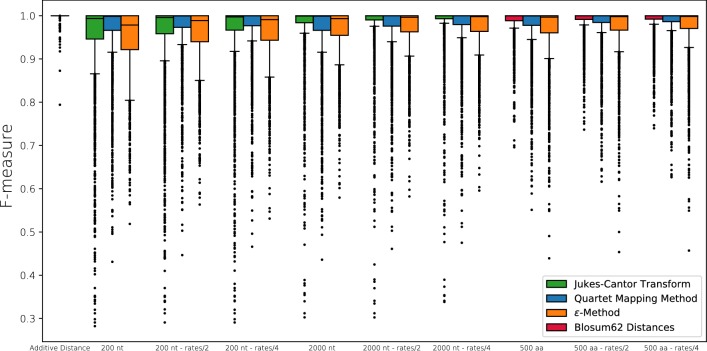
Comparison of the quality of best match estimates in terms of the F-measure for short nucleic acid sequences (200 nt), long nucleic acid sequences (2000 nt), and amino acid sequences (500 aa)

Figure 9 summarizes the results for simulated nucleic acid and amino acid sequences of different lengths and different scaling of the evolutionary rates. As expected, the short sequences incur a relative large noise level compared to the perfect additive distances. Nevertheless, the overwhelming majority of best matches is still estimated correctly (*F*-measures well above 0.9 for the vast majority of scenarios even for nucleic acid sequences as short as 200 nt). Larger false positive rates are observed only in a small number of scenarios with many duplications and losses. This is not surprising since our relatively simple rule for outgroup choice tends to fail if there are many ancient duplications. As expected, the F-measure improves with increasing sequence length due to the increased amount of information from which the distances are estimated. Since the standard deviation of the estimated distances (normalized by sequence length) decreases $\sim n^{-1/2}$, the main effect of the sequence length is to tune the noise level. Likewise, the F-measure improves when saturation effects are reduced by down-scaling the total number of events. To this end the edge lengths in the original trees were rescaled by a factor of 1/2 and 1/4, respectively. We expect, however, that reducing the number of even events further will ultimately lead to a decreasing performance, since deriving topology information from almost identical sequences is difficult or even impossible. The same trends were observed for simulated protein sequences.

Figure 9 also shows that the Quartet Mapping method outperforms the other methods for the 200 nucleotide sequences, indicating that this approach is advantageous when sequence length is small. In case of long nucleic acid sequences (2000 nt) and amino acid sequences (500 nt), the best results are obtained by estimating additive distances from pairwise sequence comparison using the Jukes-Cantor transform or a BLOSUM-based transformation, respectively.

The rather disappointing performance of the QM method for long sequences is probably the consequence of the majority voting procedure used choose the quartets. We suspect that majority voting is too simple-minded in situations where none of the three possible splits dominates. In the default setting, these are interpreted as unresolved trees ($\times$) and inserted as bi-directional edges into the auxiliary graph $\Gamma$. This, however, leads to a moderate overprediction of best matches. Alternatively, a consensus can be taken over multiple choices of the outgroup *z*. Finally, the unresolved quartets can be omitted altogether in the construction of the auxiliary graph $\Gamma$. Both alternatives perform worse than the default method, see Additional file 1: Fig. S8.

**Fig. 10:**
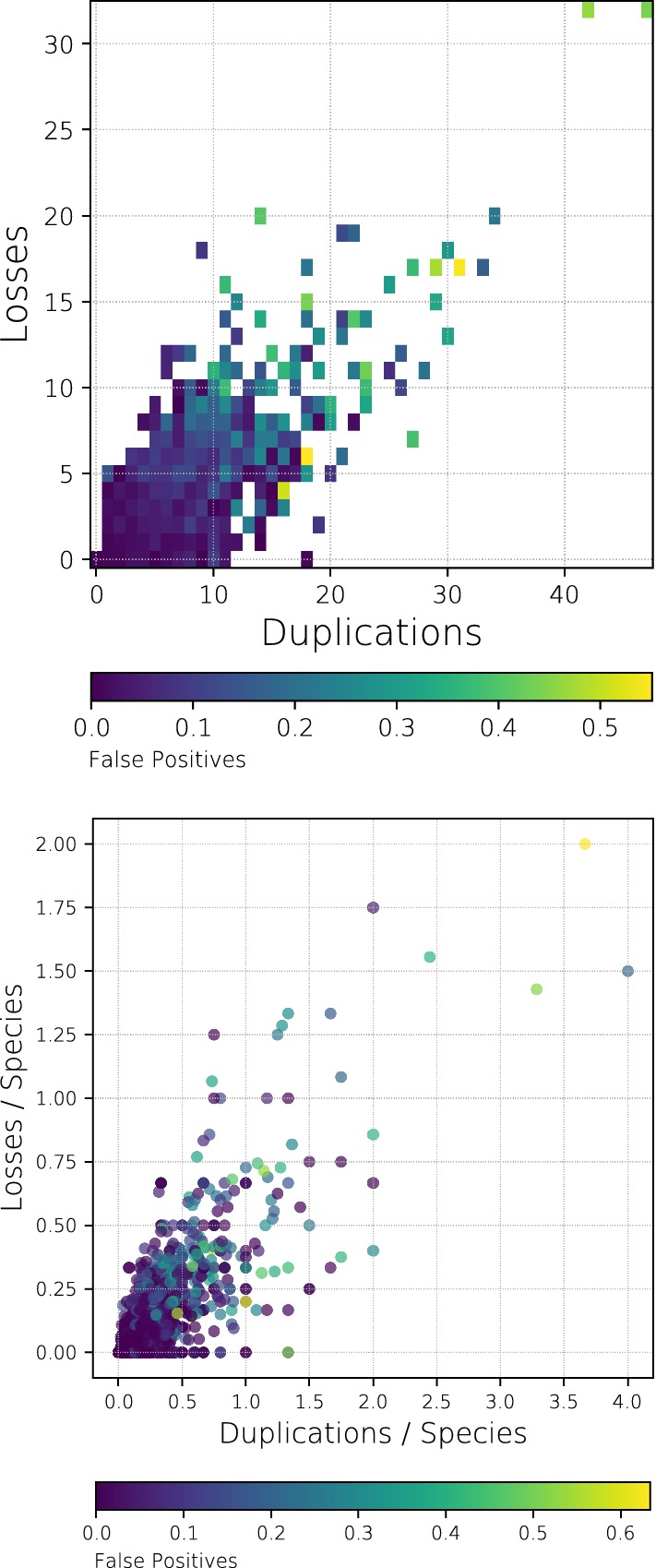
Inference of best matches from simulated sequence data (2000 nt sequences). Heat map of the fraction of false positive best matches inferred by QM as a function of the number of duplication and loss events in the simulated scenario. Upper panel: absolute number of events; lower panel: number of events normalized by the number of species. The false positive rate is computed relative to the number of true best matches

We also investigated to what extent the number of gene duplication and losses in a scenario influences the inference of best matches. As a representative example, Fig. 10 shows the false positive rate for QM. As expected, the number of false positives increases with increasing number of duplication and loss events. This can be observed both for the absolute and the relative number of events , i.e., after normalizing by the number of species (see also Additional file 1: Fig. S9 and S10 for the 200 nt sequences and for F-measure instead of false positive rate, respectively).

## Discussion and conclusions

The idea to use quartet structures for improvement of orthology estimates is not new; it was used e.g. in QuartetS [58]. Quartets are also used as witnesses of non-orthology in OMA [59] to avoid some types of false-positives. Here, we systematically investigate how and when quartets help to improve and/or correct empirical best-hit data to identify best matches in the sense of closest evolutionary relatives. We propose that reciprocal best matches, rather than the uncorrected reciprocal best hits, should then be used to infer orthology relationships. This second step has been the topic of a separate manuscript [40], in which the mathematical connections between (reciprocal) best matches and orthology are elucidated in detail.

The key observation of the present contribution is that the best matches of a gene *x* in the set *Y* of genes from a different species can be computed correctly if for every $y',y''\in Y$ one can find a gene *z* from a third species that is an outgroup for $\{x,y',y''\}$. From a theoretical point of view, this condition is closely related to rooting the gene tree. The second necessary ingredient is an estimate of an additive evolutionary distance between the genes that is accurate enough to correctly identify the topology of a certain subset of quartets. We emphasize that this is a much less stringent condition compared to the ability of reconstructing the complete gene tree *T*.

Empirically, we observe that (partial) knowledge of the species tree (more precisely: reliable monophyletic groups) is very useful for the choice of outgroup genes *z*: excellent results are obtained by choosing a candidate *z* from a species that is an outgroup for $\sigma (x)$ and $\sigma (Y)$. The results can be further improved by using filtering criteria that identify ancient duplication events and by computing a consensus over several choices of *z*. In data sets with little measurement noise, we indeed obtain nearly perfect best match estimates. The theoretical considerations outlined here also suggest additional in-roads for further improvements by means of identifying ancient duplications, which not only serve as “witnesses of non-orthology” but can also be used to prune the set of candidate outgroups.

In order to make the methods described here applicable to very large real-life data sets, it will be necessary to optimize the computational performance. To this end, we will develop heuristic rules to prune the set *Y* in the case of large gene families. An obvious candidate is to use the $\epsilon$-method as an initial filter, where $\epsilon$ is now chosen to optimize the tradeoff between |*Y*| and false negative predictions of best matches. We expect that the heuristic rules for choosing the set *Z* of candidate outgroups can also be improved substantially.

Best matches are rarely if ever of interest in isolation. Instead, they are an intermediate construction, in particular in orthology detection or the assessment of synteny. It is difficult therefore to benchmark the translation of reciprocal best hits to reciprocal best matches in a truly realistic setting because best hit data themselves are burdened with diverse sources of errors, including incorrect sequence assembly, incorrect or missing annotation of coding sequences, and the use of different splicing isoforms. The benchmarking results shown here thus have to be taken with a grain of salt. In particular, we expect that error levels of a pipeline that determines best hits and then converts them to best matches will be dominated by the first step, i.e., the computation of best hits from genome or proteome data. Our data also show, however, that there are difficult instances for which we currently have no good way to compute the correct best matches. Fortunately, these appear to be rare.

We expect that methods for orthology assessment can be improved in both reliability and computational performance by combining the accurate estimation of best matches described here with a better understanding of (reciprocal) best match graphs [38, 39, 60] and their connection with the orthology relation [40]. Since tree-free methods for orthology detection rely on (pairwise) best hits as proxy for reciprocal best matches, we expect that the accuracy of most tools would improve if best matches are supplied as input data. This is not easy to test, however, since the best hit computation is usually an integral part of the software. Such a benchmark study is hence beyond the scope of this contribution.

The work reported here is primarily intended to provide a solid theoretical foundation for the construction of improved best match heuristics. The theoretical results give some guarantees for obtaining the correct best matches and highlight some limitations that cannot be overcome with certainty as long as only distance data are available. The most promising additional data source is synteny, or more precisely, genomic proximity [61]. Given two proximal genes *u* and *v* from different families in species *A* and a pair of family members $u'$ and $v'$ proximal in species *B*, it is very likely that either both $u,u'$ and $v,v'$ or neither of them are best matches. A more systematic development of such filters will be the topic of future work.

The software used for simulating and testing the conversion of best hits to best matches has been made available on github [62]. As a next step, it will be incorporated into ProteinOrtho [50, 63, 64] to assess and benchmark the achievable improvements on real-life data. Best matches instead of best hits could of course also be used in other orthology detection tools.

## Methods

### Simulations of *dated* species trees

As in previous work [40], we use the Innovation Model [65] to produce realistic topologies for the planted species tree *S*. We then construct a dating function $\tau :V(S)\rightarrow [0,1]$ such that $\tau (0_S)=1$ and $\tau (x)=0$ for $x\in L(S)$. In order to assign a date to an interior vertex, we traverse *S* top-down, more precisely for the current node *u* at time $\tau (u)$ we proceed as follows:

1. We pick a child $v\in \mathsf {child}(u)$ and a leaf $x\in L(S(v))$ in the subtree below *v*. If *v* is already a leaf, we set $\tau (v)=0$ and proceed to the next child of *u*.
2. Otherwise, we determine the number *k* of speciations on the path between *v* and *x*. Hence, the path from *u* to *x* comprises $k+2$ edges.
3. We pick a random number *r* with mean 1 and range (0, 2) from a uniform distribution and set $\tau (v) = \tau (u) (1-r/(k+2))$. This rule is chosen so that the expected time elapsed along the edge *uv* equals $\tau (u)$ divided by the number $(k+2)$ of edges along the path to the root and ensures that $\tau (v)>0$. The result is a dated species tree in which each edge *uv* has length $\tau (u)-\tau (v)$.

The choice of the uniform distribution in (3) is a mere convenience. In principle it should be replaced by an empirically estimated distribution. Alternatively, generators capable of producing dated trees such as TreeSimGM [66] could be used.

### Simulation of gene trees in the dated species tree

We use the Gillespie algorithm [67] to simulate the duplication, loss and horizontal gene transfer events (HGT) occurring in *S*. The branches of the species tree *S* are independent in Duplication/Loss scenarios. However, horizontal gene transfer introduces dependencies between them. We therefore have to simulate the evolution process in such a way that at each time point $\tau$ the possible reactions are given by the Cartesian product $G(\tau )\times \{D,L,H\}$, where $g\in G(\tau )$ is a gene that is present at time $\tau$ in any one of the branches of the dates species tree, and $q\in \{D,L,H\}$ is one of the three possible events (Duplication, Loss, HGT). Every possible simulation event $\xi :=(g,q)$ is associated with a rate $r_{\xi }(\tau )$ that may depend explicitly on the point in time. Rate constants are described below.

In each step, two random numbers $r_1$ and $r_2$ are drawn independently from the uniform distribution on [0, 1]. The first random number $r_1$ is used to select $\xi$ with probability $r_{\xi }(\tau )/R(\tau )$, where $R(\tau )$ is the sum of the rates of all reactions available at time $\tau$. We refer to [67] for a convenient way to implement the rate-proportional choice of the “reaction channel”. Depending on the selected event type, the following actions are performed:

($q=L$) Gene loss is modeled by removing *g* from the list of active genes.

($q=D$) Gene duplications are modeled by placing a copy $g'$ of *g* into the same branch of *S* at time $\tau$.

($q=H$) For HGT the copy of $g'$ is placed into a different branch of *S*. The “landing site” for the HGT copy is chosen uniformly from the branches of *S* available at time $\tau$ with the exception of the branch harboring the parental gene *g*.

The rules determining the rate parameters for gene copies $g'$ and the optional adjustment of rates for the genes *g* are discussed below. The second random variable $r_2$ is used to update the clock according to $\tau \leftarrow \tau -\Delta \tau$ with $\Delta \tau =\ln (1/r_2)/R$. The simulation terminates as soon as $\tau -\Delta \tau \le 0$.

A complication arises from the fact that the time interval $[\tau ,\tau -\Delta \tau ]$ may contain a speciation event at time $\tau _s$. At a speciation, the gene content is copied into the daughter-lineages, and the rates are modified in a lineage-specific manner. As a consequence, the waiting time $\Delta \tau$ has to be re-estimated since the set of reaction channels has changed. More precisely, we need to determine the distribution of waiting times from a time point $t_0$ until the next event conditioned on the fact that no event occurred between $t_0$ and $t_1$, where $t_1$ designates the time point of the speciation. For the complementary cumulative distribution function and $s :=t_0-t_1$ we have

$$\begin{aligned}&{\mathbb {P}}(T \ge s+t | T \ge s) \\&\quad = {\mathbb {P}}(T \ge s+t \wedge T \ge s)/{\mathbb {P}}(T\ge s) \\&\quad = {\mathbb {P}}(T \ge s + t)/{\mathbb {P}}(T \ge s) \end{aligned}$$

Since the waiting time distributions are exponential with rate $r_1$ before $t_1$ and rate $r_2$ following the speciation event, we obtain

$$\begin{aligned} {\mathbb {P}}(T \ge s+t | T \ge s) = e^{-(r_1 s + r_2 t)}/e^{-r_1 s} = e^{-r_2t} \end{aligned}$$

Hence, if the simulated waiting time reaches beyond the speciation event, the clock is advanced to the speciation event and a new waiting time is drawn with the rates after the speciation event. In practice, a new random number to obtain the time step $\Delta \tau '$ with the updated rates after the speciation event. In the new interval $[\tau _S,\tau _S-\Delta \tau ']$ we again have to check for speciation events. Since the speciation events are known *a priori* from the dated species tree *S*, they are held in a priority queue in temporal order. The final result is a dated gene tree *T*, i.e., each event is unambiguously associated with a time stamp. The simulation also completely determines the reconciliation map $\mu$.

We simulated 2000 pairs of species and gene trees, where |*L*(*S*)| was drawn uniformly from the interval [3, 50]. The duplication and loss rates were (independently) drawn from [0.5, 1.0).

### Modeling rate imbalances

In order to produce realistic (sequence) data, an evolution rate $\omega _e$ has to be associated with each edge *e* of *T*. To this end we use a hierarchical model that first determines a baseline gene substitution rate $\omega ^0_e$ for each edge *e* of the species tree *S* in order to simulate effects such as variations of population size and generation time. This introduces a correlation between the rates of all genes in the same lineage of *S*. These base rates are then modified by gene-specific contributions that capture effects such as differences in selection pressures that depend on gene function and rate differences in the wake of duplications such as neofunctionalization and subfunctionalization [15]. In detail, we use the following parametrization:

- mean substitution rate of the conserved members of a gene family (default 1.0).
- variance $\sigma _0^2$ for the baseline substitution rate in *S* (default 0.2).
- a gamma distribution for the substitution rates $>1$ of divergent genes. The parameters are estimated from data for the whole genome duplication in saccharomycete yeasts [52]. Alternatively, a uniform distribution on $(1; r_{max}]$ can be selected.
- weights for the relative frequency of the possible fates of duplicates (functional conservation, subfunctionalization, neofunctionalization; default equal weights 1/3).

We determine the baseline substitution rates $\omega _{uv}^0$ for the edge $uv \in E(S)$ as follows: We simply assign the mean substitution rate to the planted edge $0_S\rho _S$ (i.e. 1.0 by default). We traverse *S* in pre-order and draw for each edge $uv \in E(S) \setminus \{0_S\rho _S\}$ the logarithm $\ln \omega _{uv}^0$ of the rate of evolution from a normal distribution with variance $\sigma ^2 = \sigma _0^2(\tau (u)-\tau (v))$. To avoid bias towards higher or lower rates, we normalize the mean of the normal distribution such that $E(\omega _{uv}^0 )= \omega _{\mathsf {par}(u)u}^0$.

For the gene specific rates we first sort all vertices $u \in V(T)$ by $\tau (u)$ in descending temporal order. We keep track of the current number of genes in each branch of the species tree. During the simulation, the edges of *T* will be marked as either conserved or divergent depending on the fate of the branch after a duplication event. For each edge $e=uv \in E(T)$ in the gene tree, we initialize an empty list ${\mathfrak {L}}_e$ of ordered pairs of the form $(\tau ,\omega )$ to record the gene-specific evolution rates $\omega$ and the corresponding time points $\tau$ at which they become valid during the existence of *e*. This allows us to reset the divergent status of a gene in case it is the last survivor in a given species. At present, we do not consider other events that change the rate of evolution of a gene within the edge *e*. The framework, however, can easily accommodate such rules in future refinements of the model. We denote by ${\mathfrak {L}}_{e,i}$ the ith ordered pair $(\tau _i,\omega _i)$ in ${\mathfrak {L}}_e$ and define $\tau ({\mathfrak {L}}_{e,i}) :=\tau _i$ and $\omega ({\mathfrak {L}}_{e,i}) :=\omega _i$.

Recall that $0_T\rho _T$ is the first (planted) edge in *T*. To initialize the simulation, we mark $0_T\rho _T$ as conserved and append $(\tau (0_T), 1.0)$ to ${\mathfrak {L}}_{0_T\rho _T}$. Then for each vertex *u* in the sorted list we proceed as follows:

1. *u* is a speciation event
  Mark all edges *uv* with $v \in \mathsf {child}(u)$ the same as $\mathsf {par}(u)u$. To ${\mathfrak {L}}_{uv}$ we append the pair $(\tau (u), \omega )$ with $\omega =1.0$ (*uv* is conserved) or $\omega$ Gamma-distributed (*uv* is divergent), respectively.
2. *u* is a duplication event
  If the edge $\mathsf {par}(u)u$ is marked as divergent, then all edges *uv* with $v \in \mathsf {child}(u)$ are also marked as divergent and corresponding pairs $(\tau (u),\omega )$ are appended to ${\mathfrak {L}}_{uv}$, where the values of $\omega$ are drawn i.i.d. from the Gamma distribution.
  If $\mathsf {par}(u)u$ is marked as conserved, we choose between (a) conservation, (b) subfunctionalization and (c) neofunctionalization with the specified weights. For (a) mark both incident edges below *u* as conserved, for (b) as divergent and for (c) one edge is conserved and the other is divergent. To ${\mathfrak {L}}_{uv}$ we append the pair $(\tau (u), \omega )$ with $\omega =1.0$ (*uv* is conserved) or $\omega$ Gamma-distributed (*uv* is divergent), respectively.
3. *u* is a loss event
  If a single copy is left in the respective species after the loss: Let $e^*$ be the corresponding edge of the remaining copy at $\tau (u)$. Mark $e^*$ as conserved and append the pair $(\tau (u),1.0)$ to ${\mathfrak {L}}_{e^*}$.
4. *u* is an HGT event
  Let $v_1$ be the copy that remains in the species and $v_2$ the transferred copy. Mark $uv_1$ the same as $\mathsf {par}(u)u$ and append $(\tau (u),\omega )$ to ${\mathfrak {L}}_{uv_1}$ where $\omega$ is the last rate that was appended to ${\mathfrak {L}}_{\mathsf {par}(u)u}$. Mark $uv_2$ as divergent and append $(\tau (u),\omega )$ to ${\mathfrak {L}}_{uv_2}$ with $\omega$ Gamma-distributed.
  For each edge $e=uv$ in *T* we finalize ${\mathfrak {L}}_{e}$ by appending $(\tau (v),\omega )$ where $\omega$ is the last rate that was appended to ${\mathfrak {L}}_{e}$ so far. We then define the edge length $\ell (e)$ for each edge *e* in *T* as
  6 $$\begin{aligned} \ell (e) = \omega _{f}^0 \sum _{i=1}^{|{\mathfrak {L}}_{e}|-1}\omega ({\mathfrak {L}}_{e,i}) \cdot (\tau ({\mathfrak {L}}_{e,i}) - \tau ({\mathfrak {L}}_{e,i+1})) \end{aligned}$$
  where *f* is the edge in the species tree *S* into which *e* is embedded.

### Computation of distances

The resulting function $\ell : E(T)\rightarrow {\mathbb {R}}^+$ (see Eq. 6) defines an additive metric on the set of vertices *V*(*T*). We denote by *d* a distance function on the set of non-loss leaves in *T* (i.e., the extant genes at time $\tau =0$), representing the evolutionary distance between each pair of these genes. In order to compute *d*, we first construct the *observable* gene tree ${\tilde{T}}$ by removing all branches that lead to losses only, and then contracting all inner vertices that are left with a single child. The distance *d*(*x*, *y*) of two leaves *x* and *y* in ${\tilde{T}}$ is given by the sum of edge lengths on the unique path $P_{xy}$ connecting *x* and *y* in ${\tilde{T}}$, thus

7 $$\begin{aligned} d(x,y) = \sum _{e\in P_{xy}} \ell (e). \end{aligned}$$

### Simulation of measurement noise

In order to simulate measurement noise we consider three strategies:

1. Adding i.i.d. random noise to the additive distance *d* in general violates the triangle inequality, i.e., the condition $d(x,y)\le d(x,z)+d(z,y)$ no longer holds for all $x,y,z\in L$. We therefore use the following simple algorithm: choose two distinct $x,y\in L$ at random. Moreover, we draw a noise factor $\varepsilon _{xy}$ from a normal distribution with mean 1 and standard deviation *s*, then substitute the distance of *x* and *y*, i.e. *d*(*x*, *y*) and *d*(*y*, *x*), by $d':=\varepsilon _{xy}d(x,y)$. If the perturbed distance $d'$ satisfies the triangle inequality, we accept the perturbed distance $d'$. Otherwise, $d'$ is rejected and a new random perturbation is generated. We repeat this until $$\left( {\begin{array}{c}|L|\\ 2\end{array}}\right)$$ perturbations have been accepted. An alternative approach is to first introduce perturbations to all distances and then to extract a corrected distance ${{\hat{d}}}$ using one of several algorithms for the “metric repair problem”, see e.g. [68, 69]. A cursory test showed that the trees reconstructed from distance matrices processed with these methods tend to be more different from the reference than with our approach of enforcing the triangle inequality immediately. We therefore did not pursue them further in this contribution.
2. We denote by ${\mathbf {D}}$ a distance matrix on the set of non-loss leaves in *T* whose entries correspond to the distances of *d*. It is easy to see that a convex combination $(1-\alpha ){\mathbf {D}} + \alpha \mathbf {D'}$, $0\le \alpha \le 1$ of two metrics ${\mathbf {D}}$ and $\mathbf {D'}$ is again a metric (i.e., in particular, satisfies the triangle inequality). Even if both ${\mathbf {D}}$ and $\mathbf {D'}$ are additive, however, their convex combination is not additive in general. This yields a distance that is affected by a systematic bias corresponding to the noise contribution $\alpha \mathbf {D'}$.
3. The edge lengths $\ell (e)$ (see Eq. (6)) can also be interpreted as the average number of evolutionary events per site. These are then simulated directly using the generation tool pyvolve [70]. We generated nucleic acid sequences of length 200 with equal transition and transversion rates and of length 2000 with a transition : transversion ratio of 2 : 1. To assess saturation effects, we scaled the rates $\mu _e$ by 1/4, 1/2, 1, and 2, respectively. To better connect this work with protein-based orthology assessment pipelines, we simulated aminoacid sequences of length 500 using the WAG model [71]. Distances were then estimated in Biopython [56] with the BLOSUM62 matrix [72] for aminoacid sequences and with the Jukes-Cantor model for nucleid acid sequences.

### Quartet mapping

In order to estimate quartets directly from aligned sequence data, we use the approach of statistical geometry [73, 74]. We start from a multiple alignment of the sequences *x*, $y'$, $y''$, and *z*, which we assume to appear in this order. The alignment itself is produced by the sequence simulator and thus does not need to be recomputed. Each alignment column belongs to one of the 15 categories determined by which of the four sequence *x*, $y'$, $y''$, and *z* feature the same character:

**Table:** 

	${\mathscr {C}}_{1}$	${\mathscr {C}}_{2}$	${\mathscr {C}}_{3}$	${\mathscr {C}}_{4}$	${\mathscr {C}}_{5}$	${\mathscr {C}}_{6}$	${\mathscr {C}}_{7}$	${\mathscr {C}}_{8}$	${\mathscr {C}}_{9}$	${\mathscr {C}}_{10}$	${\mathscr {C}}_{11}$	${\mathscr {C}}_{12}$	${\mathscr {C}}_{13}$	${\mathscr {C}}_{14}$	${\mathscr {C}}_{15}$
*x*	*a*	*a*	*a*	*a*	*b*	*a*	*a*	*a*	*a*	*a*	*a*	*b*	*b*	*b*	*a*
$y'$	*a*	*a*	*a*	*b*	*a*	*a*	*b*	*b*	*a*	*b*	*b*	*a*	*a*	*c*	*b*
$y''$	*a*	*a*	*b*	*a*	*a*	*b*	*a*	*b*	*b*	*a*	*c*	*a*	*c*	*a*	*c*
*z*	*a*	*b*	*a*	*a*	*a*	*b*	*b*	*a*	*c*	*c*	*a*	*c*	*a*	*a*	*d*

The categories ${\mathscr {C}}_{1}$ through ${\mathscr {C}}_{5}$ and ${\mathscr {C}}_{15}$ do not convey phylogenetic information. Of the remaining ones, ${\mathscr {C}}_{6}$, ${\mathscr {C}}_{9}$, and ${\mathscr {C}}_{14}$ support $(xy'|y''z)$, ${\mathscr {C}}_{7}$, ${\mathscr {C}}_{10}$, and ${\mathscr {C}}_{13}$ support $(xy''|y'z)$, and ${\mathscr {C}}_{8}$, ${\mathscr {C}}_{11}$, and ${\mathscr {C}}_{12}$ support $(xz|y'y'')$ [57]. Denoting by $d_{aaaa}$, etc., the number of alignment columns belonging to a given category, the support scores for *quartet mapping* (also referred to as *geometry mapping*) [57] are

8 $$\begin{aligned} \begin{aligned} S(xy'|y''z)&= d_{aabb} + \frac{1}{2}(d_{aabc}+d_{bcaa}) \\ S(xy''|y'z)&= d_{abab} + \frac{1}{2}(d_{abac}+d_{baca}) \\ S(xz|y'y'')&= d_{abba} + \frac{1}{2}(d_{abca}+d_{baac}) \\ \end{aligned} \end{aligned}$$

Using $S:=S(xy'|y''z)+S(xy''|y'z)+S(xz|y'y'')$, normalized scores are defined as $s(xy'|y''z):=S(xy'|y''z)/S$. This unweighted version can be extended to a weighted version when a non-trivial distance measure *D* on the underlying alphabet is given. As derived in [57], a support value for the three possible quartets can be computed separately for each alignment column *i* as the isolation index for the distances on the four characters:

9 $$\begin{aligned} \begin{aligned} 2\beta _i(xy'|y''z)&= D^*_i - (D(x_i,y_i') + D(y_i'',z_i)) \\ 2\beta _i(xy''|y'z)&= D^*_i - (D(x_i,y_i'') + D(y_i',z_i)) \\ 2\beta _i(xz|y'y'')&= D^*_i - (D(x_i,z_i) + D(y_i',y_i'')) \\ \end{aligned} \end{aligned}$$

Here $D_i^*$ is the largest of the three distance sums appearing in Eq. (9). Summing up the $\beta _i(\,.\,)$ values over all alignment columns *i* yields aggregated support scores $\beta (\,.\,)$. These are conveniently normalized to relative values as in the unweighted case. The relative support scores for the weighted model reduce to the unweighted ones if $D(a,b)=1-\delta -{a,b}$ is the trivial metric [57]. If no quartet can be inferred unambiguously, then we default to the assumption ${{\,\mathrm{lca}\,}}(x,y')={{\,\mathrm{lca}\,}}(x,y'')$.

## Supplementary information

**Additional file 1.** This file contains the additional figures that are referenced in the text, including an example gene tree with distances, various statistics of the simulated data set, and the additional results.

**Additional file 2.** This archive contains the simulated data set comprising 2000 species and gene tree scenarios (trees.zip), the current version of the AsymmeTree package (v0.0.5) for the simulation of such weighted scenarios. Moreover, the Python scripts for the generation and analysis of sequence data are supplied (see README.txt for more details).

## Data Availability

Software implementing most of the tasks and workflows described in this contribution is available in the AsymmeTree library for the simulation and analysis of phylogenetic scenarios https://github.com/david-schaller/AsymmeTree. We do not provide a sequence generator since third-party tools have been used for this purpose. The simulated evolutionary scenarios used throughout this contribution are available as Additional file 2.
