# Supplementary material for: From pairs of most similar sequences to phylogenetic best matches
Source: Algorithms Mol Biol. 2020 Apr 9;15:5. doi: 10.1186/s13015-020-00165-2 (PMC7147060; doi:10.1186/s13015-020-00165-2)
Supplement: Supplementary file 1 — Additional file 1. This file contains the additional figures that are referenced in the text, including an example gene tree with distances, various statistics of the simulated data set, and the additional results. [file 13015_2020_165_MOESM1_ESM.pdf]

From pairs of most similar sequences to phylogenetic best matches

Additional Figures

Peter F. Stadler, Manuela Geiß, David Schaller, Alitzel López Sánchez, Marcos González Laffitte, Dulce I. Valdivia, Marc Hellmuth, Maribel Hernández Rosales

April 6, 2020

Example of simulated scenario

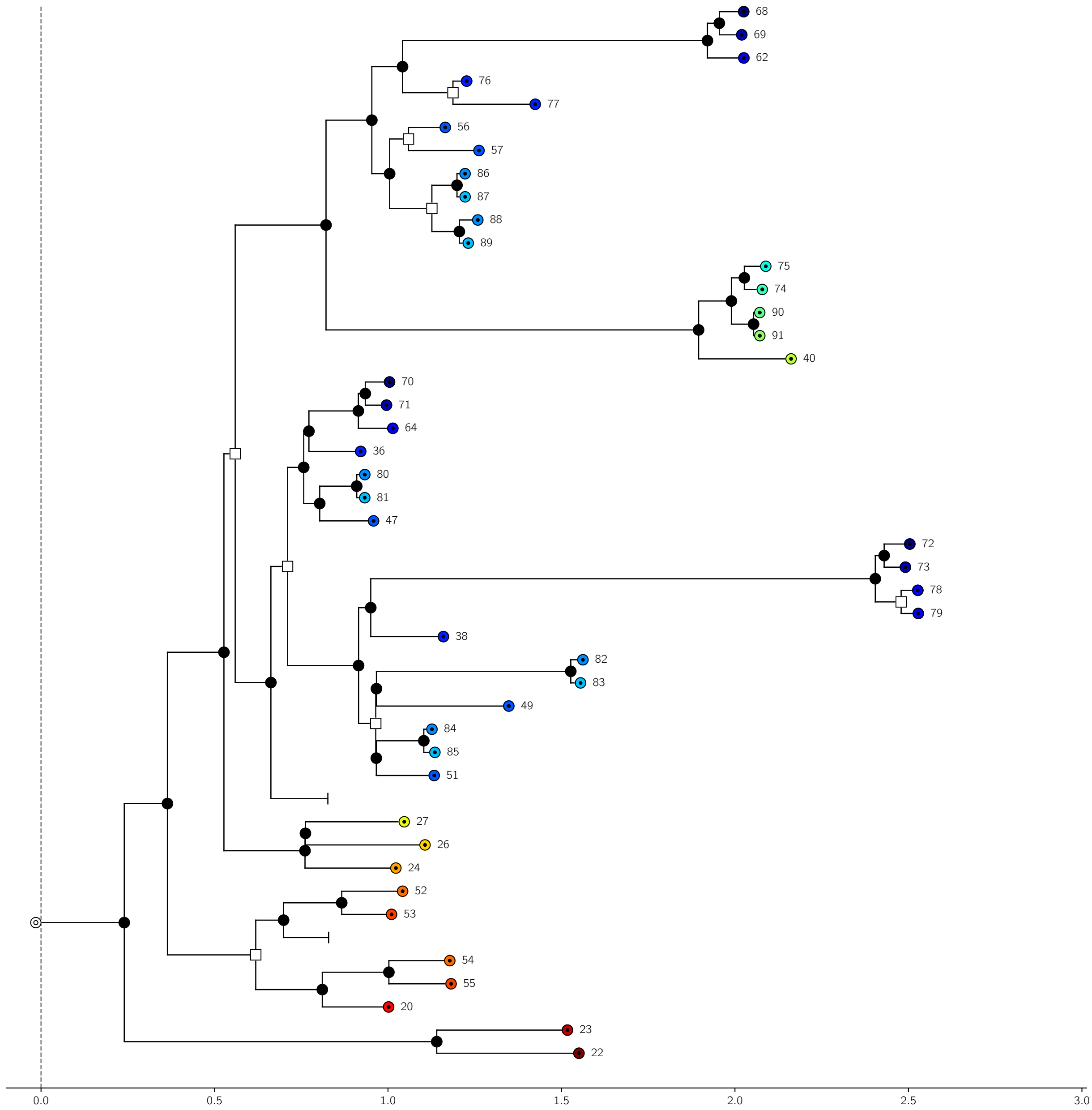

Figure S1: Example gene tree (`simID_4`) with 44 suriving leaves in 20 different species (color-coded).

Statistical properties of the 2000 simulated scenarios

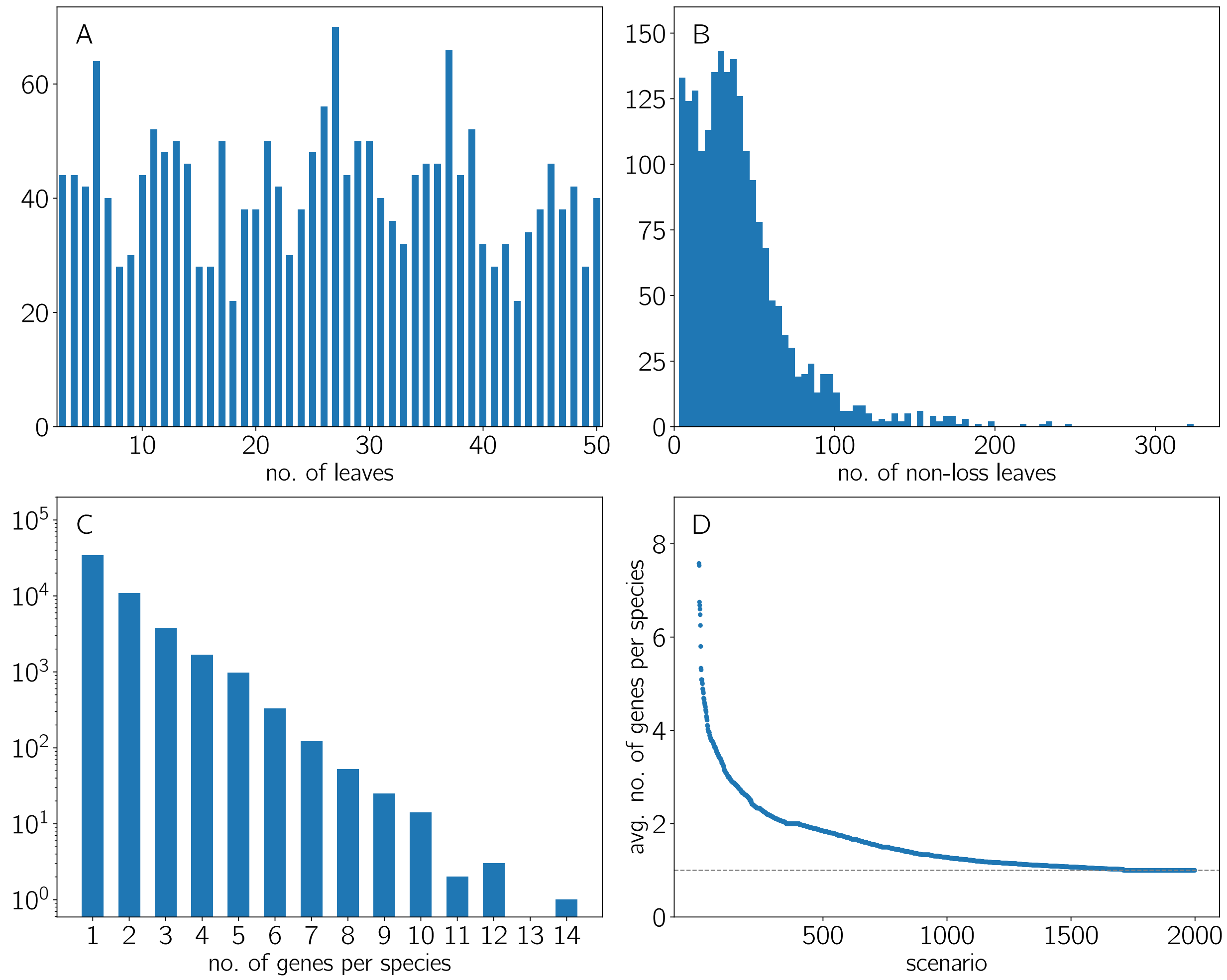

Figure S2: Distribution of the number of leaves in the set of 2000 simulated scenarios: (A) species trees (barplot), (B) (observable part of the) gene trees (histogram, 80 bins), (C) number of genes per species in the whole data set (barplot, log-scale), and (D) average number of genes per species per scenario (in descending order).

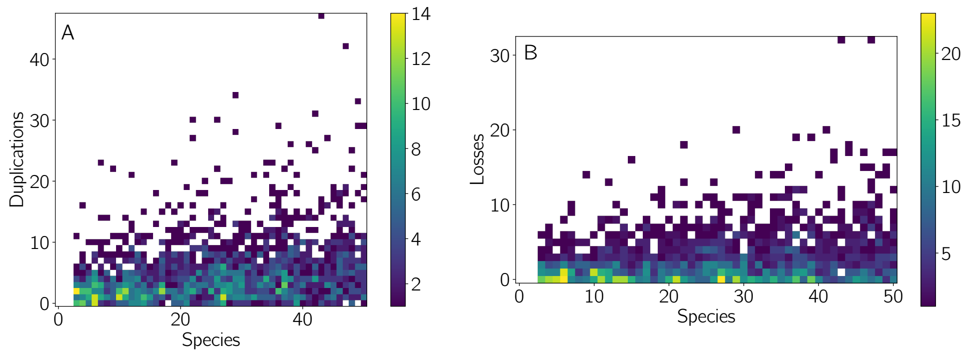

Figure S3: Distribution of the number of (A) leaves in the species tree and duplication events, and (B) leaves in the species tree and loss events as color-coded 2-dimensional histograms.

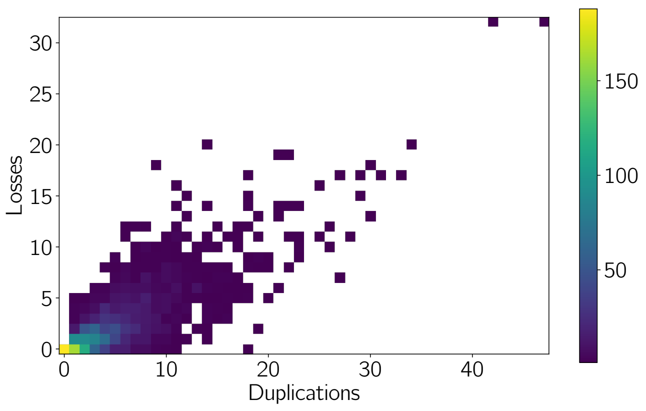

Figure S4: Distribution of the number of duplication and loss events as a color-coded 2-dimensional histogram.

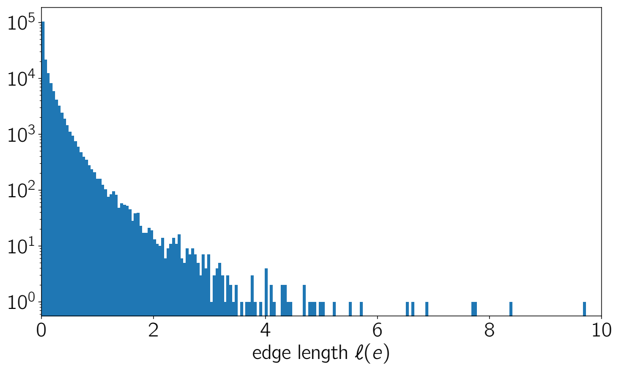

Figure S5: Distribution of the edge lengths in the simulated gene trees (histogram, 200 equal-sized bins, log-scale).

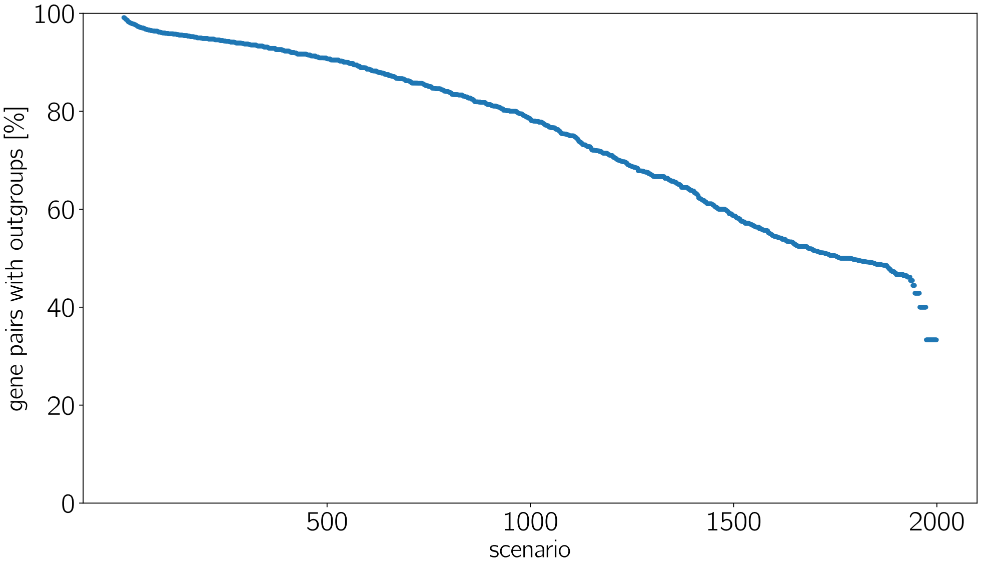

Figure S6: Percentage of gene pairs for with outgroup genes could be found (based on the heuristic that uses outgroup species) among all $n(n-1)/2$ gene pairs per scenario, where n is the number of non-loss leaves. These gene pairs with available outgroups were used to calculate recall and precision for the comparison of the best match inference methods.

Additional results

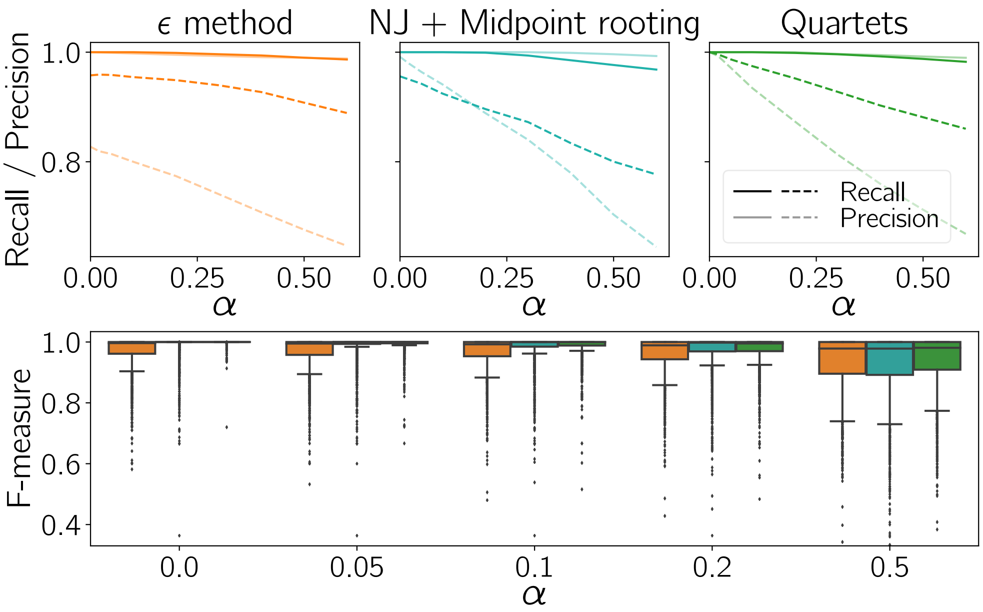

Figure S7: Performance comparison of the best match inference from distance data for simulated data (2000 scenarios) and biased noise. Top panel: Median (solid) and 10th percentile (dashed) of recall and precision as a function of noise level α, i.e., the contribution of an additive disturbance matrix $\mathbf{D}'$ that was built from another tree. For each gene tree and noise level, the final distance matrix was computed as $(1-\alpha)\mathbf{D} + \alpha\mathbf{D}'$ (see Simulation of measurement noise section). Lower panel: Boxplots of F-measure for different levels of noise superimposed on the additive distance; $\alpha = 0$ refers to perfect data. Orange: ϵ method, turquoise: explicit construction of the unrooted tree $\overline{T}$ and midpoint rooting, green: inference of quartets with outgroups chosen in another branch of the root.

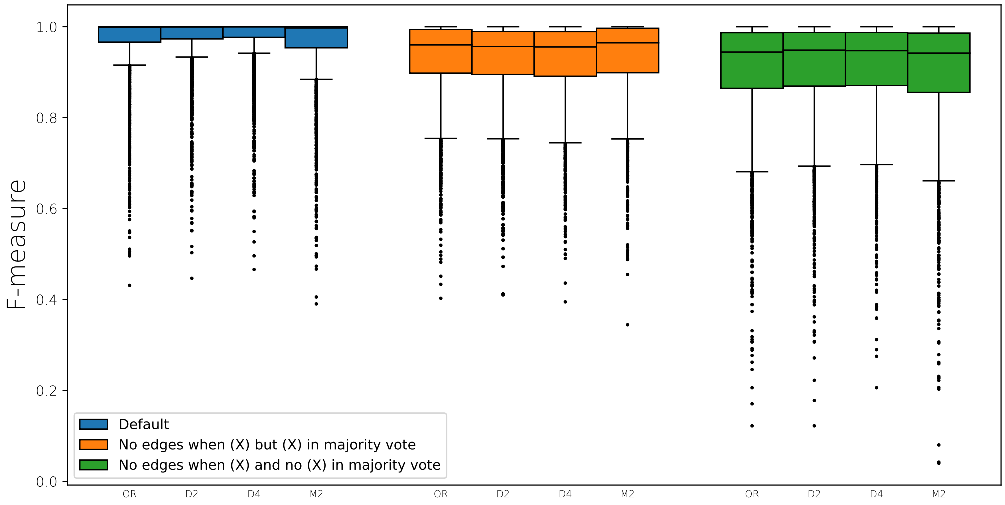

Figure S8: Alternative construction of Γ. In order to further investigate the inaccuracies introduced by unresolved quartets, we considered alternative constructions of the auxiliary graph Γ. In addition to the default method, we omitted all edges defined for quartets classified as unresolved ($\times$), and we ignored the contribution of outgroups that lead to unresolved and used a majority vote only for the remaining choices of the outgroup. All non-trivial sinks in Γ were then interpreted as best matches, i.e., isolated vertices in Γ were ignored. Both variants perform worse than the default method. Data are compared for short nucleic acid sequences with rates of sequence divergence scaled by 1 (OR), 1/2 (D2), 1/4 (D4), and 2 (M2).

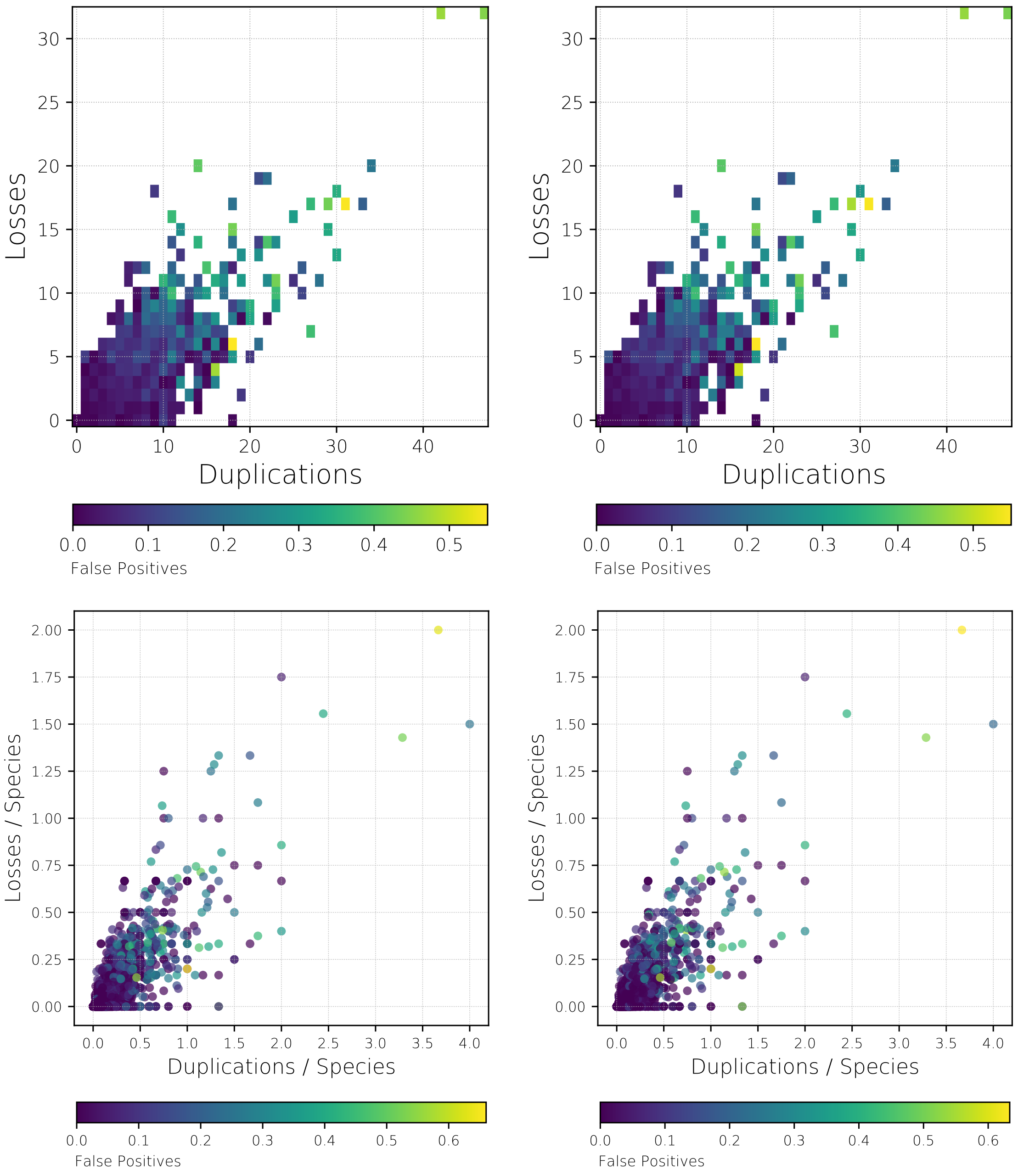

Figure S9: Inference of best matches from simulated sequence data. Heat map of the fraction of false positive best matches inferred by Quartet Mapping as a function of the number of duplication and loss events in the simulated scenario. Upper panels: absolute number of events; lower panel: number of events normalized by the number of species. Left panels: 200 nt sequences; right panels: 2000 nt sequences.

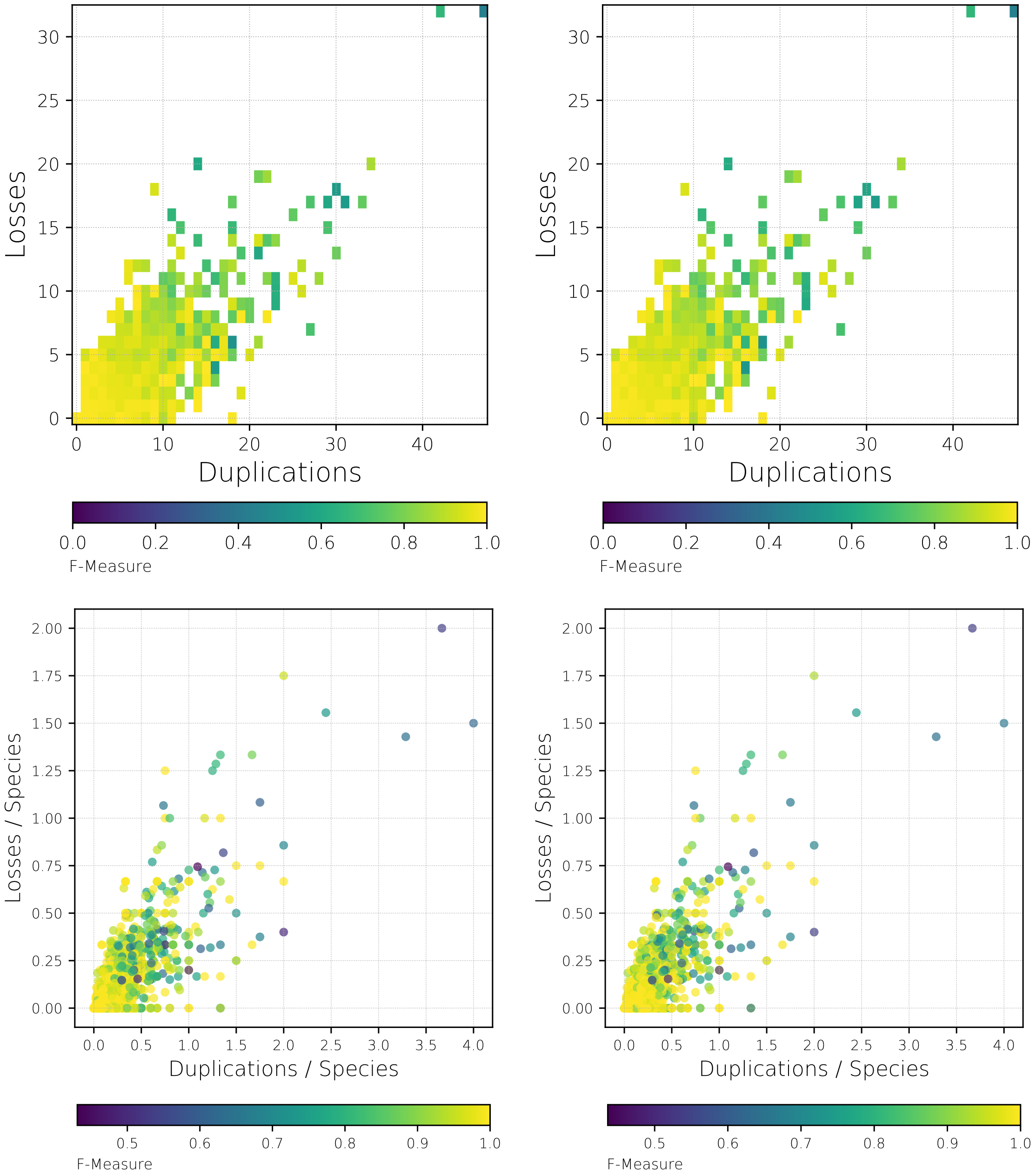

Figure S10: Inference of best matches from simulated sequence data. Heat map of the F-measure obtained using Quartet Mapping as a function of the number of duplication and loss events in the simulated scenario. Upper panels: absolute number of events; lower panel: number of events normalized by the number of species. Left panels: 200 nt sequences; right panels: 2000 nt sequences.
